# LY6D marks pre-existing resistant basosquamous tumor subpopulations

**DOI:** 10.1038/s41467-022-35020-y

**Published:** 2022-12-06

**Authors:** Daniel Haensel, Sadhana Gaddam, Nancy Y. Li, Fernanda Gonzalez, Tiffany Patel, Jeffrey M. Cloutier, Kavita Y. Sarin, Jean Y. Tang, Kerri E. Rieger, Sumaira Z. Aasi, Anthony E. Oro

**Affiliations:** 1 Program in Epithelial Biology, Stanford University School of Medicine, Stanford, CA USA; 2 Department of Pathology, Stanford University School of Medicine, Stanford, CA USA; 3 Department of Dermatology, Stanford University School of Medicine, Stanford, CA USA

**Keywords:** Predictive markers, Basal cell carcinoma, Tumour heterogeneity

## Abstract

Improved response to canonical therapies requires a mechanistic understanding of dynamic tumor heterogeneity by identifying discrete cellular populations with enhanced cellular plasticity. We have previously demonstrated distinct resistance mechanisms in skin basal cell carcinomas, but a comprehensive understanding of the cellular states and markers associated with these populations remains poorly understood. Here we identify a pre-existing resistant cellular population in naive basal cell carcinoma tumors marked by the surface marker LY6D. LY6D^+^ tumor cells are spatially localized and possess basal cell carcinoma and squamous cell carcinoma-like features. Using computational tools, organoids, and spatial tools, we show that LY6D^+^ basosquamous cells represent a persister population lying on a central node along the skin lineage-associated spectrum of epithelial states with local environmental and applied therapies determining the kinetics of accumulation. Surprisingly, LY6D^+^ basosquamous populations exist in many epithelial tumors, such as pancreatic adenocarcinomas, which have poor outcomes. Overall, our results identify the resistant LY6D^+^ basosquamous population as an important clinical target and suggest strategies for future therapeutic approaches to target them.

## Abstract (web summary)

The identification of distinct cell populations with cellular plasticity in skin basal cell carcinoma is important for understanding treatment resistance mechanisms. Here, the authors identify the resistant LY6D^+^ basosquamous population that correlates with poor clinical outcomes.

## Introduction

Targeted therapies have proven beneficial in treating cancers, but dynamic tumor heterogeneity and an array of pre-existing and induced resistance mechanisms act to circumvent treatment. Growing evidence points to inherent cellular plasticity or phenotype switching as one of the most challenging resistance mechanisms. While epithelial stem cell plasticity confers a remarkable ability of cells to toggle their behaviors and cellular identities, drivers of changing phenotypes and markers for populations that correlate with poor clinical outcomes are currently lacking^1^.

Non-melanoma skin cancers or keratocarcinomas such as basal cell carcinomas (BCCs) and squamous cell carcinomas (SCCs) represent ideal model systems to study dynamic tumor heterogeneity and plasticity because of their accessibility for serial biopsies and the well-defined lineage relationships within the skin^2,3^. BCC growth depends on the canonical activation of the Hedgehog (HH) signaling pathway, primarily driven through various mutations in genes such as Patched (*PTCH1*) or the G-protein coupled receptor Smoothened (*SMO*)^4^. While HH pathway inhibitors targeting SMO (SMO^i^) are the primary therapy in advanced non-surgically resectable cases, 60% are already resistant^2^. Furthermore, acquired resistance occurs in ~20% of patients who initially respond to SMO^i^, reinforcing the need to delineate tumor evolution mechanisms^2^.

Although distinct in their molecular drivers, clinical observations point to a fluid relationship between BCCs and SCCs, which have clear implications for treatment strategies. First, it is routinely observed that BCCs treated with SMO^i^ can develop resistance in the form of a transition towards SCCs after being re-biopsied^5,6^. In contrast to BCCs, which exhibit features of the hair follicle (HF) epithelium progenitors, SCCs are not HH-driven and resemble the interfollicular epidermis (IFE)^2,3^. In line with this clinical observation, some BCC mouse models point to a SMO^i^-dependent lineage transition resistance mechanism, producing a persister population of cells characterized by loss of HH dependence and toggling towards a differentiated IFE-like cell fate^7^. Second, the existence of sporadic basosquamous carcinomas (BSCs), which show features of both BCCs and SCCs, further points to the presence of a basosquamous transition (BST) in keratocarcinomas^8,9^. Further investigation found that the SCC portions of BSCs are BCC-derived but lose their dependence on HH signaling^9^. With both sporadic and induced observations, BST etiology remains complex, with various drivers and inducers such as SMO^i^, transcriptional inducers like c-FOS, or subsequent mutations such as ARID1A^5,6,9,10^.

A critical unresolved question is whether populations that undergo BST originate from the selection of small pre-existing resistant populations or are induced in response to the therapy. It is unclear what the relationship is between these observed populations, the mechanisms that drive their accumulation, the key markers that define and track them, and whether they exist in other epithelial tumors.

Here we deconvolute BST mechanisms utilizing the identified stem cell surface marker Lymphocyte Antigen 6 Family Member D (LY6D), which marks and tracks basosquamous populations. LY6D marks the SMO^i^-induced IFE-like resistant population, but importantly, it also marks a pre-existing population in naive human sporadic BCCs. We show that LY6D^+^ tumor cells lie on a differentiation spectrum between BCC and SCCs, and resemble basosquamous cell carcinoma (BSC), highlighting pathway switching as a cellular plasticity-mediated resistance mechanism. Further, we show how SMO^i^ expedites the transition towards the LY6D^+^ state and demonstrate that the LY6D^+^ population marker can identify other basosquamous states in epithelial tumors with poor outcomes.

## Results

### LY6D marks a pre-existing spatially localized basosquamous BCC population

**Fig. 1:**
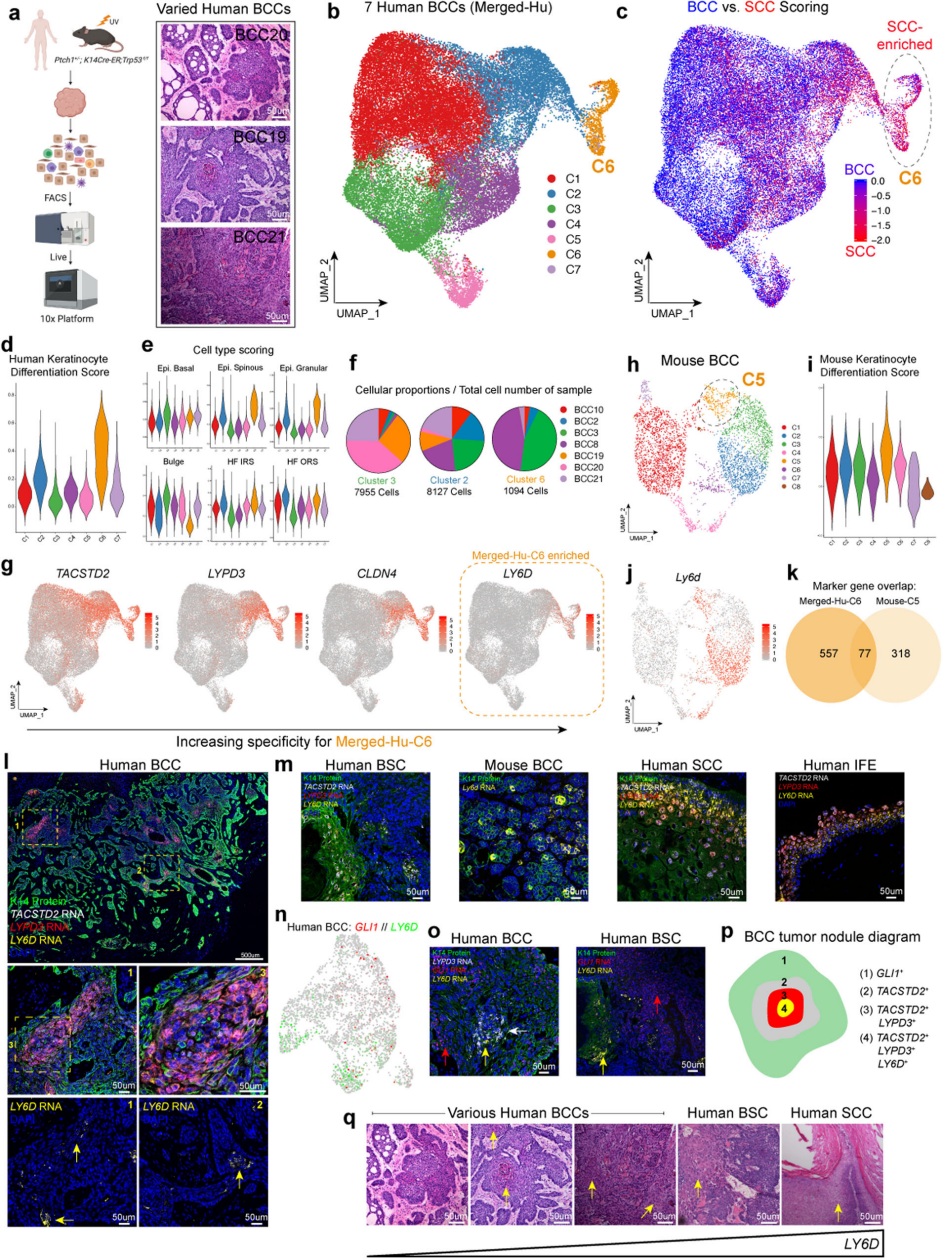
LY6D marks a pre-existing spatially localized basosquamous BCC population. **a** scRNA-Seq study design (left) and H/E of the various histological features from different human BCCs used for scRNA-Seq experiments. Scale bar = 50 μm. *n* = 7 humans and *n* = 1 mouse. **b** UMAP plot of scRNA-Seq data from seven merged human BCC tumor epithelial samples with seven distinct clusters. **c** BCC vs SCC scoring superimposed onto the UMAP plot from **b**. Merged-Hu-C6 from **b** is circled. **d** Gene scoring for Keratinocyte Differentiation GO term for the seven different clusters from **b**. **e** Cell type scoring for various skin epithelial cell types for the seven different clusters from (**b**). **f** Tumor sample proportions in clusters Merged-Hu-C3, Merged-Hu-C2, and Merged-Hu-C6. **g** Feature plots highlighting the expression of key surface markers (*TACSTD2*, *LYPD3*, *CLDN4*, and *LY6D*) with the specificity of *LY6D* for Merged-Hu-C6 indicated. **h** UMAP plot of scRNA-Seq data from mouse BCC tumor epithelial sample with eight distinct clusters. **i** Gene scoring for Keratinocyte Differentiation Score for the eight different clusters from **h**. **j** Feature plot of *Ly6d* in mouse BCC tumor. **k** Marker gene overlap between Merged-Hu-C6 and Mouse-C5. **l** Spatial localization of *TACSTD2* (white), *LYPD3* (red), and *LY6D* (yellow) transcripts in human BCCs, which are co-stained with K14 protein (green). *LY6D* expression is also shown alone without other markers. Zoomed regions indicated by yellow boxes. Yellow arrows point to *LY6D*^+^ regions. *n* = 3. **m** Spatial localizations of basosquamous markers in human BSC, mouse BCC, human SCC, and human IFE. The markers shown are *TACSTD2* (white), *LYPD3* (red), and *LY6D/Ly6d* (yellow). *n* = 2 for human examples. *n* = 3 for mouse. **n** Feature plot human BCC21 showing expression of *GLI1* and *LY6D*. **o** Spatial localization of basosquamous state and *GLI1* transcripts in naive human BCCs and BSCs. Red arrows point to *GLI1*^*+*^ regions. Yellow (*LY6D*^*+*^) and white (*LYPD3*^+^) arrows point to basosquamous regions. *n* = 2. **p** Diagram indicating the spatial localizations of the basosquamous markers with respect to *GLI1*^+^ cells. **q** Histology of various human BCCs, BSC, and SCC with the corresponding indication of *LY6D* levels. Yellow arrows point to squamous features. Length of each scale bar is noted in figure.

Our approach to deconvolute BST-associated plasticity aimed to define populations and markers within BCCs, identify potential signs of BST, and then interrogate factors that promote interconversion. We generated a comprehensive dataset by merging seven existing and new (BCC19, BCC20, and BCC21) datasets from naive human BCC tumors with varying histological features and then used appropriate canonical correlation analysis (CCA) to mitigate patient-to-patient variability (Fig. 1a, b, Supplementary Fig. 1a–c, e)^11^. CopyKAT, which specifically differentiates tumor and normal tissue via copy number variations (CNVs), validated our ability to discriminate between tumor and normal tissue (Supplementary Fig. 1d)^12^. As a result, we found seven distinct clusters, each with its unique marker genes and corresponding Gene Ontology (GO) associated terms (Fig. 1b, Supplementary Fig. 1e–h, Supplementary Data 1).

Surprisingly, the naive BCC dataset already contained SCC-like resistant populations arising from BST. We used BCC and SCC gene signatures to score the different cellular populations and found a spectrum of BCC- and SCC-like states among the different clusters where C6 from our merged dataset (Merged-Hu-C6) had significant enrichment for SCC-associated gene signatures, identifying the cluster of SCC-like tumors’ pre-existence in naive BCCs (Fig. 1b, c)^10^. Consistent with the SCC-like state, Merged-Hu-C6 was enriched for epidermis-related GO terms, keratinocyte differentiation gene scoring, and differentiated related cell types such as spinous and granular IFE but not the HH responsive HF epithelium (Fig. 1d, e, Supplementary Fig. 1h–i)^13^. This observation suggests that merged-Hu-C6 represents an intermediate state between BCC and SCC, resembling a BSC/basosquamous state, which may be resistant to SMO^i^^9^. To assess the incidence of this population in naive BCCs, we interrogated the presence of the Merged-Hu-C6 population in all seven naive BCCs. We found the basosquamous population present in all, albeit with varied frequency, supporting the commonality of this pre-existing population (Fig. 1f). Furthermore, we noted that other clusters representing intermediate SCC-like clusters with an enriched for (Cluster 2) and non-SCC-like (Cluster 3) were also distributed in all seven naive BCCs, suggesting a spectrum of tumor cell states (Fig. 1f).

To prospectively isolate the Merged-Hu-C6 basosquamous population, we used highly expressed marker genes specific to Merged-Hu-C6 with subsequent verification by COMET to probe our scRNA-Seq for associated surface markers and identified *LY6D, CLDN4*, *LYPD3*, and *TACSTD2* (Fig. 1g, Supplementary Fig. 1j)^14^. We have found the surface markers interchangeable, especially with subsequent staining experiments, but found that *LY6D* had the greatest Merged-Hu-C6 specificity (Fig. 1g). LY6D is part of the Ly6 family of lymphocyte lineage and stem cell markers that have roles in marking solid organ lineages as well^15^. Notably, *LY6D* expression is also enriched in the well-differentiated human SCC cluster and marks the overlap between human BCC and SCC scRNA-Seq data and the IFE suprabasal cells of human foreskin scRNA-Seq data, further drawing a parallel to the basosquamous cellular state observed in naive human BCCs (Supplementary Fig. 2a–g)^16,17^. We then examined naive murine BCCs from the *Ptch1*^*+/−*^*;K14-creER;p53*^*fl/fl*^ strain to determine whether the same marker could be used in mouse tumor biology (Fig. 1a, h). Highlighting its utility, *Ly6d* also marked the basosquamous population in naive murine BCC tumors (Fig. 1j). Although multiple clusters expressed *Ly6d*, perhaps suggesting increased *Ly6d* correlates with larger and more advanced tumors (a point we will return to), only C5 (Mouse-C5) expressed all basosquamous markers (*Ly6d*, *Cldn4*, *Lypd3*, and *Tacstd2*) and was the most enriched for IFE/keratinocyte differentiation-related genes (Fig. 1h–j, Supplementary Fig. 2h, i, Supplementary Data 2). Additionally, the intersection between the human Merged-Hu-C6 and mouse Mouse-C5 clusters reveals a 77 gene set that includes *LY6D*, suggesting a similar conserved basosquamous state across species (Fig. 1k; Supplementary Data 3). We conclude that *LY6D*/*Ly6d* marks the basosquamous population in human and mouse tumors.

Using our surface markers as basosquamous readouts, we found that the populations occupy unique spatial localizations within the tumor. RNAScope delineated the population, with *LY6D* marking the cells in the most central portions of tumor nodules (Fig. 1l, Supplementary Fig. 2j). Like the suprabasal cells in the normal human epidermis, these clusters of cells were noticeably separated from the basement membrane zone (BMZ) and did not interface with the stroma (Fig. 1l, Supplementary Fig. 2j). Mouse BCCs had similar patterns with *Ly6d* expression found centrally localized in nodules (Fig. 1m). Extensive expression was also observed in well-differentiated human SCCs and the more SCC-like portions of BSCs compared to the BCC-like regions (Fig. 1m).

We next interrogated the status of the HH signaling pathway in LY6D^+^ basosquamous cells by checking whether *LY6D*^+^ cells overlapped with the pathway target gene *GLI1*. In line with the previous finding that Merged-Hu-C6 was not enriched for HH-related genes, *LY6D* and the associated surface markers failed to colocalize with *GLI1* (Fig. 1n, Supplementary Figs. 1i, 2k). Additionally, scATAC-Seq (which we will discuss at length later) lineage trajectories suggested an inverse relationship between *GLI1* and *LY6D* (Supplementary Fig. 2l). Finally, we confirmed by RNAScope that *LY6D*^+^ clusters in both naive BCCs and BSCs do not colocalize with *GLI1* (Fig. 1o).

Overall, we conclude that LY6D marks a previously unidentified spatially localized basosquamous-like population lacking GLI signaling, which is present before SMO^i^ treatment. This population lacks *GLI1* expression, is spatially localized, and is demarcated by *TACSTD2*, *LYPD3*, and most specifically by *LY6D* (Fig. 1p). Our analysis of multiple naive BCCs, BSCs, and SCCs suggests levels of *LY6D* can vary from tumor to tumor but dramatically increase along the BST spectrum towards SCC (Fig. 1q).

### BCCs transition towards an LY6D^+^ state while maintaining proliferative capacity

**Fig. 2:**
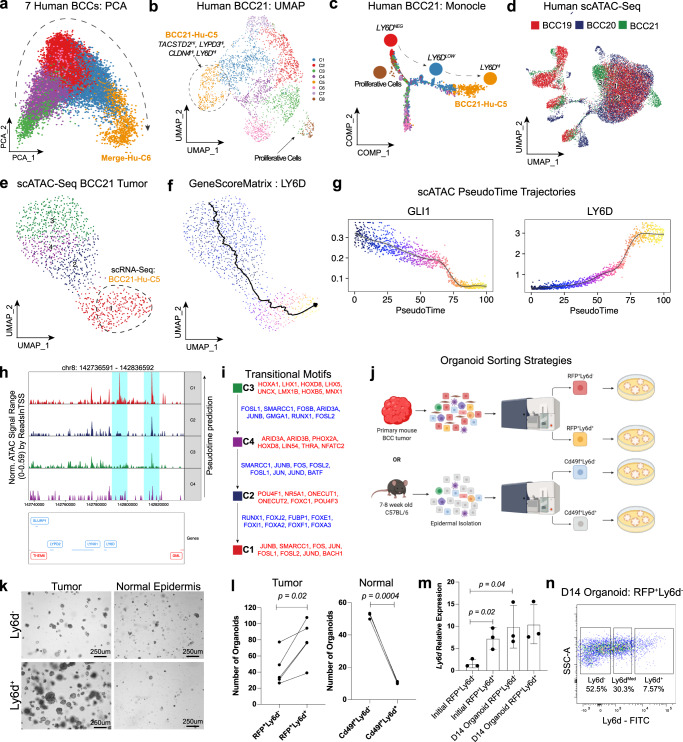
BCCs spontaneously transition towards an LY6D^+^ state. **a** PCA projection of the seven merged human BCC tumor epithelial samples from Fig. 1b. Arrow indicates presumptive lineage transition. **b** UMAP plot of scRNA-Seq data from human BCC21 tumor epithelial sample with eight distinct clusters. The circled BCC21-Hu-C5 population, is enriched for indicated markers. **c** Monocle projection of human BCC21 tumor epithelial sample with the same cluster information from Fig. 2b. Arrow indicates transition from an *LY6D*^*Neg*^ to BCC21-Hu-C5. **d** UMAP plot of scATAC-Seq data from three merged human three BCC tumor samples. **e** UMAP plot of scATAC-Seq data from human BCC21 tumor epithelial sample with four distinct clusters. **f** Feature plot of the GeneScoreMatrix for LY6D for human BCC21 scATAC-Seq data as shown in Fig. 2e. Monocle projection overlayed the predicted lineage transition. **g** scATAC-Seq lineage trajectories for GLI1 and LY6D. **h** Chromatin accessibility for around LY6D locus for each cluster from human BCC21 scATAC-Seq. LY6D is on the negative strand (genes in blue). Peaks enriched for the scATAC-Seq C1/red cluster are indicated in blue. **i** Motifs enriched in the open regions of each cluster (red) and motifs involved in the transitions between clusters (blue). **j** Sorting strategies from tumors or mouse skin used for organoid cultures. **k** Brightfield images of organoids grown from Ly6d^+^ or Ly6d^−^ epithelial cells isolated from either mouse BCCs or mouse epidermis. **l** Quantification of the number of organoids from BCCs and normal epidermis. Each point represents number of organoids from either BCCs (*n* = 5) or mouse skin (*n* = 3). **m** *Ly6d* expression from Ly6d^+^ or Ly6d^−^ BCCs directly after sorting or from Ly6d^+^ or Ly6d^−^ tumor epithelial cells cultured as organoids. Each point represents *Ly6d* expression level normalized to *Gapdh* from distinct (*n* = 3) primary tumors from different mice. n. Flow analysis of Ly6d^−^ BCCs cultured as organoids for 14 days. Length of each scale bar is noted in figure. **m** Error bars represent mean ±SD. **l**, **m**, *p* values were calculated using unpaired, two-tailed *t* test. Source data are provided as a Source Data file.

The spatial localization of LY6D^−^ and LY6D^+^ tumor populations were of great interest, prompting further investigation into their potential relationship. We utilized several computational approaches to support the lineage progression from LY6D^−^ to LY6D^+^ tumor states. First, principal component analysis (PCA) of the merged human BCC scRNA-seq dataset indicates the *LY6D*^+^ Merged-Hu-C6 is a BCC lineage endpoint (Fig. 2a). Furthermore, PCA and Monocle analysis of BCC21 (where BCC21 C5 expresses high levels of *LY6D*, referred to as BCC21-Hu-C5) reinforce a transition from an *LY6D*^−^ state to an *LY6D*^+^ state (Fig. 2b, c, Supplementary Fig. 2l, m, Supplementary Data 4). These observations were further validated with Monocle pseudotime analysis of our mouse scRNA-Seq data, revealing the *Ly6d*^−^ to *Ly6d*^+^ transition (Supplementary Fig. 2n).

Second, single-cell ATAC-Seq (scATAC-Seq) supports sequential chromatin accessibility changes between LY6D^−^ and LY6D^+^ populations. We analyzed three human BCC tumors with accompanying scRNA-Seq data and used the ArchR algorithm to define cluster relationships (Fig. 2d, Supplementary Fig. 3a–d, Supplementary Data 5)^18^. An example analysis of the tumor epithelial subset of BCC21 found at least four distinct clusters, which varied in their chromatin accessibilities near various marker genes (Fig. 2e, Supplementary Fig 3e–k, Supplementary Data 6–8). Integration of BCC21 scRNA-Seq data identified scATAC-Seq C1 as highly similar to BCC21-Hu-C5 (Fig. 2e, Supplementary Fig. 3l). scATAC-Seq C1 showed enhanced accessibility for *LY6D* along with reduction for *GLI1*, and like the lineage predictions from the scRNA-Seq, the scATAC-Seq lineage prediction tool via ArchR predicts scATAC-Seq C1 as the endpoint (Fig. 2f, g). Looking at the scATAC-Seq tracks, we note distinct peaks associated with scATAC-Seq C1 in the promoter regions of *LY6D* (Fig. 2h). Lastly, consistent with sequential cluster transition, we note distinct transcription factor motifs appear and close (Fig. 2i, Supplementary Fig. 3m, n). Previous work has implicated the AP-1 family motifs in the transition from BCCs to SCCs^10^. We found that AP-1 family motifs were enriched at the early stages of the transition (scATAC-Seq C3 to C4 and C4 to C2), but that enriched motifs such as RUNX1 and various forkhead box family of transcription factors were involved in the regulation of the LY6D^+^ state (Fig. 2i). Our chromatin dynamics analysis strongly supports a spontaneous transition to an LY6D^+^ population.

Third, to functionally confirm that the transition from an LY6D^−^ to LY6D^+^ state is tumor cell-autonomous and spontaneous, we generated an SMOi-sensitive mouse BCC organoid system to culture bulk or sorted populations of tumor epithelial cells based on specific surface markers (Fig. 2j, Supplementary Fig. 5a–c). We sorted Ly6d^−^ cells, cultured them as organoids for 14 days, and found that after 14 days in culture, expression levels of *Ly6d* in the organoids were significantly higher compared to levels at initial plating as well as comparable to organoids from Ly6d^+^ cells grown for 14 days (Fig. 2j, k, m Supplementary Fig 4d). Additionally, when we cultured Ly6d^−^ organoids, we detected Ly6d^+^ cells by flow analysis at D14, suggesting the ability of Ly6d^−^ to transition Ly6d^−^ to the Ly6d^+^ state (Fig. 2n). Overall, this data reinforces the cell’s autonomous nature of the Ly6d^−^ to Ly6d^+^ transition. Additionally, we noted a significant growth advantage of the Ly6d^+^ organoids compared to their Ly6d^−^ counterparts (Fig. 2k, l). Importantly, this growth advantage was specific to tumor Ly6d^+^ cells as Ly6d^+^ cells from the normal epidermis were growth-arrested compared to normal Ly6d^−^ cells (Fig. 2k, l, Supplementary Fig. 5g). We conclude that despite the spontaneous suprabasal conversion from LY6D^−^ to LY6D^+^ tumor cells, LY6D^+^ BCC cells do not exit the cell cycle and maintain their proliferative potential.

### Distinct epigenetic regulation and biological processes associated with tumor epithelial cells

**Fig. 3:**
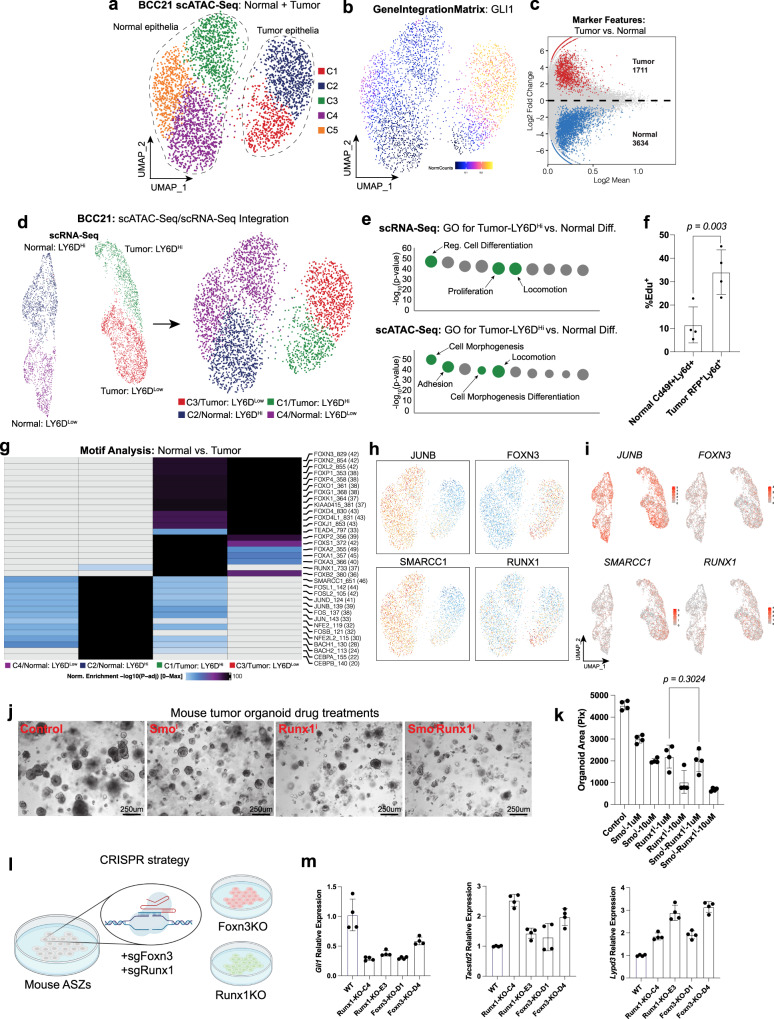
Distinct epigenetic regulation and biological processes associated with tumor epithelial cells. **a** UMAP plot of scATAC-Seq for both the tumor epithelia and normal epithelia regions within BCC21. **b** Feature plot of the GeneScoreMatrix for GLI1 for human BCC21 scATAC-Seq data as shown in Fig. 3a. **c** The differential marker features from the scATAC-Seq data of human BCC21 between tumor epithelia and normal epithelia populations. **d** UMAP plots of scRNA-Seq (left) and scATAC-Seq (right) data integration for the tumor epithelia and normal epithelia populations of human BCC21. **e** GO analysis for marker genes from (1) scRNA-Seq (top) and (2) scATAC-Seq (bottom) of C1 vs. C2. **f** Edu% of Ly6d^+^ cells from either normal mouse skin organoids or tumor organoids. Each point represents Edu% from distinct organoids (*n* = 4) derived from BCCs or normal mouse skin from different mice. **g** Heatmap indicating the enriched motifs in the various clusters from normal and BCC cells. **h** Feature plot of the MotifMatrix for various motifs identified in Fig. 3g superimposed onto the different clusters from the normal and BCC cells to indicate the specificity of enrichment. **i** Feature plot highlighting expression of the various genes (*JUNB*, *SMARCC1*, *FOXN3*, and *RUNX1*), whose motifs were identified in **h**. **j** Brightfield images of organoids from mouse BCC tumors that were treated with SMO^i^, Runx1^i^, or SMO^i^ with Runx1^i^. Inhibitor treatments were at 1 μM. **k** Quantification of the organoid area after growth with the various drugs from distinct primary tumors (*n* = 4). **l** Diagram of the CRISPR strategy to target *Foxn3* and *Runx1* in ASZs. **m** Gene expression of *Gli1*, *Tacstd2*, and *Lypd3* in wildtype and different clones of Foxn3-KO and Runx1-KO cells. Each point represents technical replicates (*n* = 4) of gene expression values of the specified gene normalized to *Gapdh*. Length of each scale bar is noted in figure. **f**, **k**, and **m** error bars represent mean ±SD. **f**, **k** *p* values were calculated using unpaired, two-tailed *t* test. Source data are provided as a Source Data file.

Because LY6D^+^ cells in the suprabasal layers of the normal IFE normally uniformly lead to proliferative arrest and terminal differentiation, we investigated the difference between the interconversion from LY6D^−^ to LY6D^+^ in both normal and BCC contexts. We took advantage of the ability to discriminate between normal and tumor cells in our human scATAC-Seq and scRNA-Seq data via chromatin accessibility and expression of *GLI1*, respectively (Fig. 3a–c, Supplementary Data 9). Using four scRNA-Seq clusters defined by both normal and BCC cells as well as LY6D^−^ and LY6D^+^ fractions (Normal: LY6D^Low^, Normal: LY6D^Hi^, Tumor: LY6D^Low^, and Tumor: LY6D^Hi^), we were able to impute and integrate these clusters onto the scATAC-Seq clustering for subsequent analysis (Fig. 3d). We would presume that the basis of these key cellular differences stem from the large difference in mutations between the tumor and normal epithelium. We had previously used CopyKat to differentiate tumor and normal epithelial tissue through CNV inferences (Supplementary Fig. 1d). Visualization of the altered CNVs between Normal: LY6D^Hi^ and Tumor: LY6D^Hi^ indicate extensive alterations in CNVs, likely reflecting the altered mutational status (Supplementary Fig. 3o)^12^.

To further understand the underlying biological processes enriched in the LY6D^+^ BCC cells compared to LY6D^+^ normal cells, we did GO analysis of differential marker genes from scRNA-Seq and peaks (in proximity to genes) from scATAC-Seq between LY6D^+^ (C1) and LY6D^−^ (C2). We identified similar terms associated with differentiation, proliferation, locomotion, and adhesion (Fig. 3e). Consistent with the observed increased proliferative differences, we noted prominent *Mki67*^*+*^*/Ly6d*^*+*^ cells in our mouse scRNA-Seq data and subsequently found Edu incorporation of Ly6d^+^ cells from the tumor are more significant than from the normal epidermis (Fig. 3f, Supplementary Fig. 5e, f).

Motif analysis of these four clusters allowed found significant enrichment for forkhead box family of transcription factors (such as FOXN3) within the BCC compared to normal cells (Fig. 3g). Interestingly, we found that although enriched in the LY6D^Hi^ BCC cells compared to the LY6D^Low^ BCC cells, the AP-1 motifs (such as JUNB) and SMARCC1 were largely specific to normal cells compared to BCC cells (Fig. 3g, h). Closer examination identified that the RUNX1 motif was uniquely enriched in the LY6D^Hi^ portion of the BCC, suggesting that this tumor-specific motif might regulate the basosquamous state transition (Fig. 3g, h). Although there was motif enrichment specificity, the expression of the identified transcription factors was largely consistent throughout the tumor and normal tissue (Fig. 3i). To functionally test whether FOXN3 and RUNX1 promoted or inhibited distinct tumor states, we performed two separate experiments. First, we tested the effects of the Runx1 molecular inhibitor (Runx1^i^) with and without Smo^i^ in our mouse tumor organoid model (Fig. 3j, Supplementary Fig. 4a). We found that both Smo^i^ and Runx1^i^ had significant effects on organoid growth, and together, the inhibitors did not display synergism, suggesting that Runx1 acts positively on BCC growth through the same downstream mechanism (Fig. 3j, k, Supplementary Fig. 4a). Second, we generated *Foxn3* (Foxn3-KO) and *Runx1* (Runx1-KO) mutant murine BCC cell lines (Fig. 3l, Supplementary Fig. 4b). Compared to wildtype controls, both Foxn3-KO and Runx1-KO cells had reduced levels of *Gli1* (Fig. 3m). Furthermore, we noted that along with the reduction in *Gli1*, we saw an increase in the expression levels of BST-associated surface markers *Lypd3* and *Tacstd2* (Fig. 3m). Together, these data support a role for Runx1 and Foxn3 in maintaining the LY6D^−^ BCC state through repression of the BST transition.

### Physical features and genetics influence LY6D^+^ cellular accumulation

**Fig. 4:**
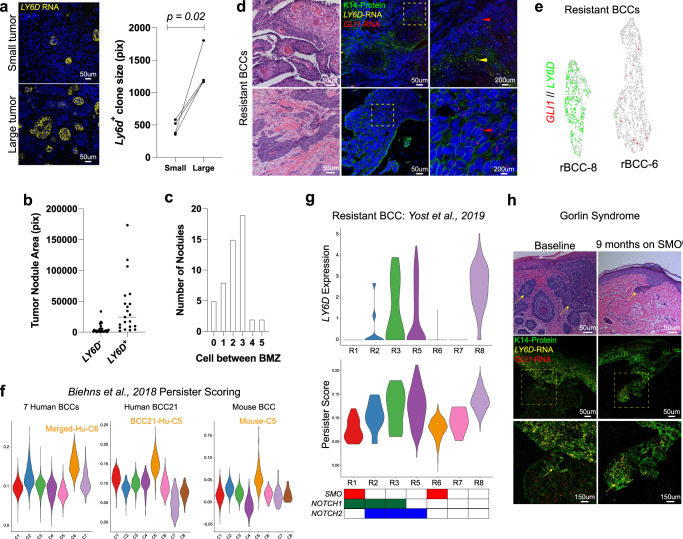
Physical features and genetics influence SMO^i^ Resistant LY6D^+^ cellular accumulation. **a** RNAScope and quantification of *Ly6d*^*+*^ (yellow) clone size from small or large tumors from the same mouse. Each point represents the average clone size from small and large tumors from the same primary mouse (*n* = 4). Lines between dots indicate the small and large tumors from the same primary tumor. **b** Tumor nodule areas from human BCC21 that are either *LY6D*^+^ or *LY6D*^−^ from Fig. 1l. The median area is indicated. **c** The number of cells between basosquamous regions and the BMZ from human BCC21 in Fig. 1l. **d** H/E and corresponding RNAScope of the spatial localization of *LY6D* (yellow) and *GLI1* (red) in resistant human BCCs, which is co-stained for K14 protein (green). The upper tumor displays an example of an rBCC with basosquamous/SCC-like features, while the lower tumor does not. Zoomed-in regions are indicated with yellow dashed boxes. Regions enriched for *LY6D* are indicated with a yellow arrow. Regions enriched for *GLI1* are indicated with a red arrow. *n* = 2. **e** Feature plot of two resistant human BCCs showing dual expression of *GLI1* and *LY6D*. R8 (left) has basosquamous markers, where R6 (right) does not. **f** Persister score (from Biehns et al., 2018) for the various BCC datasets with the Merged-Hu-C6 (left), BCC21-Hu-C5 (center), and Mouse-C5 (right) all indicated. **g** Violin plot for *LY6D* expression (top), Persister scoring (middle), and mutational status (bottom) for various resistant human BCCs (from Yost et al., 2019). **h** H/E examples of baseline (left) and SMO^i^ (right) treated tumors from Gorlin patients. Below is RNAScope indicating the spatial localization of *LY6D* (yellow) and *GLI1* (red), which is co-stained with K14 protein (green) in baseline (left) and on SMO^i^ (right) Gorlin patients. The SMO^i^ (right) treated RNAScope image is matched with the H/E above. Zoomed-in regions are indicated with yellow dashed boxes. Yellow arrows point to regions enriched for *LY6D*. *n* = 1. Length of each scale bar is noted in figure. **a** *p* values were calculated using unpaired, two-tailed *t* test. Source data are provided as a Source Data file.

While we have shown that LY6D^+^ BCCs lack HH/GLI signaling, form spontaneously without treatment, and remain proliferative compared to normal tissue, we further investigated the determinants affecting LY6D^+^ tumor cell accumulation. As previously mentioned, we noted that the bulk expression of Ly6d was higher in our mouse model compared to our human tumors. Upon quantitative analysis, we noted higher *Ly6d* bulk expression in larger mouse tumors when compared to paired smaller tumors (Fig. 4a). These results would suggest that the amount of Ly6d increases as tumors progress and get larger in size. Additionally, within BCC21, the expression of *LY6D* correlated with large nodules (Fig. 4b). Furthermore, basosquamous regions were typically found 2–3 cells away from the BMZ, suggesting that overall tumor size, intratumor nodular size, and distance from the BMZ contribute to *LY6D*/*Ly6d* levels (Fig. 4c).

Along with tumor size, drug therapy also affects the proportion of LY6D^+^ cells. When resistant BCCs (rBCCs) are re-biopsied after SMO^i^, many present extensive SCC-like phenotypes (Fig. 4d)^5^. Examining rBCCs with SCC-like features by RNAScope reveals *LY6D* expression that does not colocalize with *GLI1* (Fig. 4d). Conversely, rBCCs without SCC-like features lack *LY6D* expression but maintain *GLI1* levels throughout the tumor (Fig. 4d). Similar observations can be seen in rBCC scRNA-Seq data, with some tumors homogeneously expressing *LY6D* or *GLI1* (Fig. 4e, Supplementary Fig. 5h)^19^. Overall, this confirms that within naive BCC tumors, targeted therapy via SMO^i^ selects for LY6D^+^ populations.

Previous work has identified a persister population of cells that maintain resistance to SMO^i^, which have IFE-like characteristics^20^. We found that our LY6D^+^/Ly6d^+^ cells shared considerable overlap with this population of cells in our human and mouse naive datasets (Fig. 4f). Furthermore, rBCCs with high levels of LY6D subsequently had a high persister score (Fig. 4g)^19^. In contrast to previous observations, this data would suggest that the persister population is spontaneously generated in naive sporadic tumors, which offers a mode of BCC resistance to SMO^i^.

Not all rBCCs express *LY6D*, so we postulated that variation could be based on differential mutational landscapes that yield differential resistance. Consistent with this idea, we noted a lack of *LY6D* expression in those tumors with SMO activating mutations, irrespective of size (Fig. 4g). Additionally, we noted correlations of *LY6D* expression in rBCCs with NOTCH1 and NOTCH2 mutations, typically seen in SCCs (Fig. 4g)^9^. Patients with basal cell nevus syndrome, also known as Gorlin syndrome, have a rare heritable inactivation of *PTCH1*, predisposing them to BCCs with relatively few background mutations^21^. Clinical trial data demonstrate that while SMO^i^ therapy shrinks syndromic BCCs, they typically reform in the same location after SMO^i^ is discontinued from the persister cell population^22^. Examining naive and SMO^i^-treated tumors, we find that naive syndromic tumors express little *LY6D* but high levels of *GLI1* (Fig. 4h). By contrast, persister cell clusters in SMO^i^-treated tumors enrich for *LY6D* and lack *GLI1* expression, reinforcing the therapy dependent LY6D basosquamous state in these tumors (Fig. 4h). Altogether these observations suggest that tumors without an SMO activating mutation employ both spontaneous and drug-induced conversion strategies towards an LY6D^+^ basosquamous persister state.

### Therapeutic intervention can alter SMO^i^ Resistant LY6D^+^ cellular accumulation

**Fig. 5:**
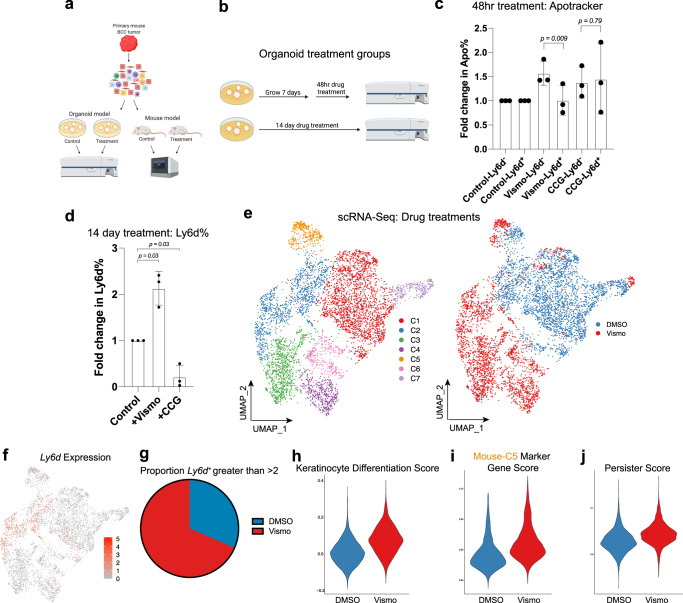
Drug treatments influence LY6D^+^ cellular accumulation. **a** Schematic diagram for tumor dissociation and subsequent experimental designs with in vitro organoid or in vivo allograft drug treatments. **b** Schematic diagram of organoid treatments designed to examine acute and long-term drug treatments. **c** Fold change in Apotracker Green in organoids treated with either SMO^i^ or CCG. Each point represents Apotracker Green% of Ly6d^−^ or Ly6d^+^ cells from distinct drug treated organoids derived from different primary tumors (*n* = 3). **d** Fold change in Ly6d% in organoids treated with either SMO^i^ or CCG. Each point represents the fold change in Ly6d% from distinct drug treated organoids derived from different primary tumors (*n* = 3). **e** UMAP plot of merged scRNA-Seq data from control and SMO^i^-treated mouse tumors by clusters (left) and treatment groups (right). **f** Feature plot for *Ly6d* expression data for Fig. 5c. **g** The proportion of cells from control or SMO^i^ treated tumors with a *Ly6d* expression level >2. **h** Keratinocyte Differentiation Score for the control or SMO^i^ treated tumors in Fig. 5c. **i** Mouse-C5 marker gene score from the mouse scRNA-Seq data for the control or SMO^i^ treated tumors in Fig. 5c. **j** Persister score for the control or SMO^i^ treated tumors in Fig. 5c. **c**, **d** Error bars represent mean +/−SD. **c**, **d** *p* values were calculated using unpaired, two-tailed *t* test. Source data are provided as a Source Data file.

To better understand how SMO^i^ and other drug interventions regulate the transition towards an LY6D^+^ state, we utilized our organoid system, plating bulk tumors and subjecting them to various inhibitors to examine how they would influence the levels of Ly6d (Fig. 5a). To test SMO^i^ selectively, we treated organoids with SMO^i^ and then checked the apoptotic levels in both Ly6d^−^ and Ly6d^+^ cells by flow cytometry (Fig. 5b, Supplementary Fig. 5i). We found enhanced cell death in the Ly6d^−^ cells suggesting that, as one might predict, SMO^i^ targeted the cells enhanced with HH signaling (Fig. 5c). Treatment with the MRTF inhibitor CCG, which has been shown to associate with the LY6D^+^ nMRTF state switching, did not show similar acute selectivity (Fig. 5c)^10,11^.

To test the long-term effect of drug intervention, we treated organoids for 14 days immediately after plating, and to test the acute effect, we treated already-formed organoids for 48 h (Fig. 5b). As predicted from our previous observations, treatment with SMO^i^ led to a modest increase in Ly6d, suggesting that long-term treatment would select for a Ly6d^+^ state (Fig. 5d; Supplementary Fig. 5j). In contrast, when organoids are treated with CCG, we see a dramatic decrease in Ly6d levels (Fig. 5d; Supplementary Fig. 5k).

To examine the global long-term in vivo effects of SMO^i^ on BCCs, we transplanted the same primary tumor and then treated the allograft tumors with either DMSO or SMO^i^ (Fig. 5a). scRNA-Seq of the treated tumors revealed distinct clustering, with enhanced *Ly6d* levels in the SMO^i^ treated tumor (Fig. 5e–f, Supplementary Data 10). In addition, the global analysis revealed enhanced differentiation and enrichment for Mu-C5 marker genes from the primary mouse scRNA-Seq (Fig. 5h, i). Finally, we noted an enrichment for the resistant persister cell state in the SMOi-treated tumor (Fig. 5j). This data supports the notion that targeted therapy through SMO^i^ can induce the LY6D^+^ persister basosquamous state.

### LY6D^+^ populations are targetable and exist in other epithelial tumors, correlating with poor clinical outcomes

**Fig. 6:**
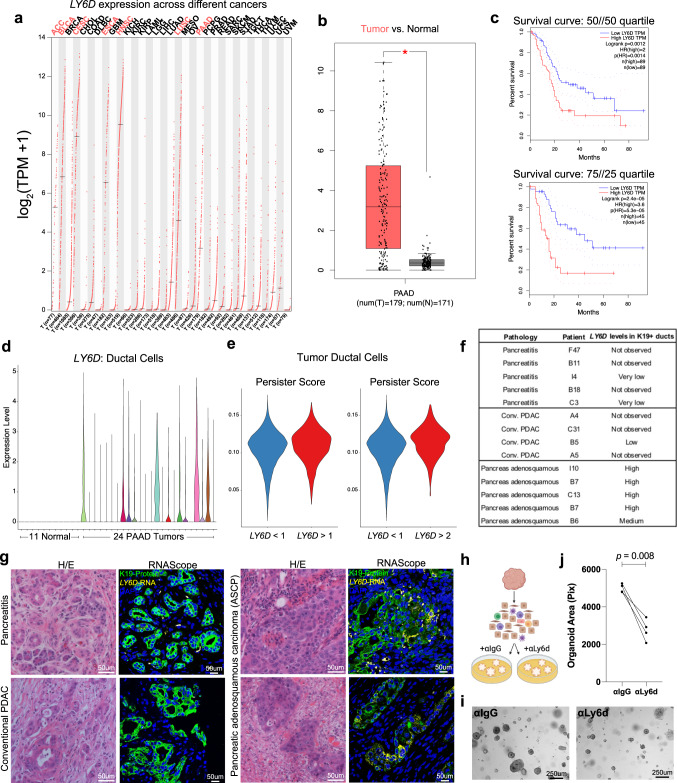
LY6D^+^ populations in epithelial tumors exhibit poor outcomes. **a** Bulk gene expression profile of *LY6D* across different cancer types. Cancers with log_2_(TPM + 1) greater than 2 are highlighted in red. **b** Bulk gene expression profile of *LY6D* between PAAD tumor and normal pancreatic tissue. 179 tumors and 171 normal samples shown. Box and whisker boundaries indicate the 25th and 75th percentile, limits indicate minima and maxima values, and center value indicating the mean. **c** Survival curves between patients with high and low levels of *LY6D* with two different quartile cutoffs. 50//50 quartile: 89 patients in high and low *LY6D* groups; 75//25 quartile: 45 patients in high and low *LY6D* groups. **d** Violin plot showing scRNA-Seq expression of *LY6D* across ductal cells from 11 control pancreas samples and 24 different tumors. **e** Persister score for tumor ductal cells that have greater than or less than a specified *LY6D* expression level. **f** Summary table of *LY6D* expression levels across multiple tumor samples. **g** H/E and corresponding RNAScope for *LY6D* (yellow) and co-stained with K19-protein (green) for human pancreatitis, conventional pancreatic adenocarcinoma (PDAC), and pancreatic adenosquamous carcinoma. *n* = 5 pancreatitis, *n* = 4 PDAC, and *n* = 5 pancreatic adenosquamous carcinoma samples. **h** Schematic diagram for mouse BCC tumor dissociation and subsequent organoid culture with and without anti-Ly6d. **i** Brightfield images of mouse BCC tumor organoids treated with anti-IgG control or anti-Ly6d. **j** Quantification of the number of organoids from BCC tumors treated with anti-IgG or anti-Ly6d. Each point represents the number of organoids from distinct primary tumors (*n* = 4). Length of each scale bar is noted in figure. **j** *p* values were calculated using unpaired, two-tailed *t* test. **b** *p* values were calculated using a one-way ANOVA with the ‘*’ indicating a *p* < 0.01. Source data are provided as a Source Data file.

Our studies indicate that LY6D distinguishes between mouse and human skin BCC- and SCC-like states, raising the possibility that it serves as a general diagnosis and treatment biomarker for other basosquamous cancers. Examining *LY6D* levels across all major cancers, we noted high levels in adenoid cystic carcinoma (ACC), bladder urothelial carcinoma (BLCA), cervical squamous cell carcinoma, and endocervical adenocarcinoma (CESC), esophageal carcinoma (ESCA), head and neck squamous cell carcinoma (HNSC), lung squamous cell carcinoma (LUSC), and pancreatic adenocarcinoma (PAAD) (Fig. 6a). Compared to normal tissue, *LY6D* levels were higher in the tumor tissue of CESC, LUSC, and PAAD (Fig. 6b, Supplementary Fig. 6a). Poor clinical outcomes were associated with high levels of *LY6D* in CESC and LUSC but dramatically higher in PAAD (Fig. 6c, Supplementary Fig. 6b).

To understand the range of *LY6D* expressed in different pancreatic tumors, we examined the ductal cells from a sizeable scRNA-Seq dataset containing both normal and tumor samples, noting a spectrum of *LY6D* expression (Fig. 6d, Supplementary Fig. 6c, d)^23^. Furthermore, we noted that tumor ductal cells with higher levels of *LY6D* had a higher persister score, raising the possibility of a similar squamous-associated state observed in BCCs (Fig. 6e).

Squamous features are sometimes noted in PAAD, referred to as adenosquamous carcinoma of the pancreas (ASCP). Reported as a generally rare pancreatic cancer event (with frequencies ranging between 0.3–10% of cases), ASCP typically has dramatically poor clinical outcomes relative to PAAD^24,25^. The frequency range stems from the difficulties in histologically identifying ASCP, indicating a clear unmet clinical need to have markers that could distinguish PAAD and ASCP. To determine whether *LY6D* is a readout for ASCP, we examined histologically defined patient tumors from conventional PAAD as well as ASCP (Fig. 6f). We found no or very low levels of *LY6D* in the K19^+^ ducts of PAAD samples or the inflamed pancreatitis state (Fig. 6f, g). However, we did find high *LY6D* expression uniformly associated with the ASCP state (Fig. 6f, g). These observations suggest that *LY6D* defines this notoriously challenging to identify ASCP tumor state.

To determine whether LY6D has a functional role beyond its clear utility as a biomarker of squamous states, we utilized our tumor organoid model and introduced anti-Ly6d antibodies (Fig. 6h). We found that the addition of anti-Ly6d antibodies led to reduced organoid size, suggesting that targeting Ly6d may, in fact, have therapeutic significance (Fig. 6h). Overall, this work highlights that *LY6D* is expressed in multiple cancers and can be targeted, suggesting that LY6D has functional relevance beyond a biomarker.

## Discussion

Using BCCs as a model system, we have identified a spontaneously occurring resistant basosquamous state marked by LY6D surface expression and show that accumulation can be enhanced or reduced by tumor mutations or drug treatment strategies. Highlighting its utility, we show *LY6D* expression in other cancers, such as the ASCP subtype of pancreatic cancer, which correlates with extremely poor clinical outcomes.

While BCC tumors can develop resistance through cell identity switching between BCC and SCC, whether the transition occurs spontaneously or after therapy remained unclear^2,9,20,26^. Identifying the basosquamous state marked by *LY6D* and lack of *GLI1* allowed us to track and characterize this resistant population, demonstrating its appearance even before SMO^i^ therapy, which may explain the high failure of monotherapy. Moreover, we unify previous observations and show the *LY6D* marks persister cells that previously were found only after SMO^i^ treatment^20^. We identify this population before SMO^i^ but show that SMO^i^ can further promote LY6D^+^ tumor accumulation. Consistent with the low SMO activity driving LY6D^+^ accumulation, human rBCCs containing high levels of SMO activity through activating mutations lacked spontaneous LY6D^+^ tumor accumulation. By contrast, Gorlin’s syndrome patients have a low base level of spontaneous LY6D^+^ cells, respond well to SMO^i^, but induce residual LY6D^+^ persister tumors that recur upon SMO^i^ cessation^21^.

It is interesting to draw parallels between the LY6D^+^ cell in BCCs and whether they can potentially serve as SCC cancer stem cells (CSCs), giving rise to the SCC-associated tumor epithelium observed in multiple contexts such as rBCCs and BSCs^27^. Data from this work and others would suggest that persister populations from Gorlin patients as well as mouse models of BCC tumor persistence enter an LY6D^+^/IFE-like state with SMO^i^ but then return to GLI1^+^/HH-dependent state after stopping therapy and not an SCC-like state^20^. This evidence would suggest that additional factors may drive cells from a BCC-associated LY6D^+^ state to an SCC-associated LY6D^+^ state with the potential to grow out an SCC-like tumor epithelium. Evidence from BSCs would suggest that additional sporadic mutations such as ARID1A are needed to make the transition to an LY6D^+^ state capable of giving rise to an SCC-associated tumor epithelium^9^. In the case of rBCCs, it has been made clear from this work that SMO^i^ can lead to an accumulation of LY6D^+^ cells suggesting that drug intervention may drive LY6D^+^ cells into a similar state capable of giving rise to an SCC-associated tumor epithelium. These data demonstrate the key role of SMO activity and background genetic mutations in the accumulation kinetics of LY6D^+^ resistant tumors and suggest kinetics may be sensitive to other therapeutic, genetic, or environmental determinants.

Our work also provides additional insights into signaling pathways that may promote LY6D^+^ BCC tumors. The central localization within tumor nodules of the basosquamous state and distance from the BMZ provides insight and speculation as to what factors may drive this intrinsic resistance. As the tumor and tumor nodules grow, the distance from the characteristic BCC tumor stroma becomes larger, suggesting that lack of stroma (its contact and/or secreted factors) may drive towards an LY6D^+^ state. Deconvoluting the contribution of the BCC stroma to general tumor growth and potential resistance has become of clear clinical importance as immunotherapy approaches are now being used to treat advanced BCCs^19^. In addition, our studies rely on previous work to identify signaling pathways that can also influence LY6D^+^ tumor accumulation^10,11^. LY6D reduction through blocking transition pathways therapeutics targeting MRTF or EGFR reduce LY6D accumulation, raising the possibility of using these drugs in concert with SMO^i^ therapy to minimize the accumulation of LY6D-associated resistance in both syndromic and sporadic BCCs. Furthermore, our work highlights the therapeutic implications of targeting LY6D itself may have functional roles, but additional considerations are needed to target tumor-specific LY6D^+^ cells.

The existence of LY6D surface expression in other epithelial cancers suggests that the work on BCCs will apply to more lethal cancers such as ASCP incidence in pancreatic adenocarcinoma. With varied suggestions on ASCP prevalence, LY6D expression can now be used to quickly identify ASCP and allow for more functional experimentation to better understand differences with PAAD. Roughly 11 of the 24 (45.8%) scRNA-Seq tumor datasets analyzed here have higher levels of *LY6D*, suggesting that ASCP might be more widespread than initially thought. Additionally, it is possible that, like the BCC-SCC epithelial plasticity spectrum, pancreatic cancer may have a more plastic nature that toggles between a non-squamous and squamous state. EGFR inhibitors, which reduced LY6D^+^ accumulation in our BCC system, are approved to treat PDAC, suggesting that they might be more effective in LY6D-expressing ACSP^10,28^. Overall, this work demonstrates the utility of LY6D as a generalizable marker of a basosquamous state with poor clinical outcomes.

## Methods

### Human samples

All patient samples were obtained through written informed consent and subsequently de-identified. All protocols for sample acquisition and usage are in accordance with the reviewed protocol by the Stanford University Institutional Review Board, protocol #18325 (Stanford, CA).

### Mice

*Ptch1*^*+/−*^*;K14-creER;p53*^*fl/fl*^ male or female mice (with or without RFP) are generated as previously indicated^26^. In short, at 7 weeks of age, recombination is induced through 3 tamoxifen injections (50 μL of 5 mg/mL; Sigma; T5648). One day after the last injection, the mice are UV irradiated at 5.25 Gy. Tumors then form ~6 months later. 8-week-old female C57BL/6 mice (Stock number: 000664) and 8-week-old female NOD SCID mice (Stock number: 001303) were obtained from the Jackson Laboratory. All mouse work was approved by the Institutional Animal Care and Use Committee (IACUC) at Stanford University, protocol #11680. Mice were fed regular chow, fed water ad libitum, and maintained under a 12-h light/dark cycle at 20–24 °C in accordance with our mouse protocol. Mouse welfare monitoring followed all experimental-dependent protocols. In accordance with our mouse protocol, the maximal tumor size/burden is 1.75 cm^3^ in volume. In our experiments, the maximal tumor size/burden was not exceeded. All mouse euthanasia followed a carbon dioxide-based euthanasia followed by cervical dislocation as described in our protocol.

### Sample processing

Human tumor specimens (BCC19, BCC20, and BCC21) were briefly rinsed with 1× PBS before being chopped and minced to pieces less than 1 mm in diameter. After mincing, tumor pieces are transferred to 50-mL conical tube and 40 mL of 0.5% collagenase solution (Gibco; 17-100-017) in DKFSM media (Gibco; 10744-019). The minced tumors are then incubated at 37 °C with rotation for 2 h. At the end of the 2-h incubation, 5 mL of 0.25% Trypsin (Gibco; 25200056) was then added to the 50-mL conical tube, which was then further incubated at 37 °C with rotation for an additional 15 min. 5 mL of FBS was then added to the cellular suspension. Mouse tumor (mBCC) specimens were processed in the same manner except for the initial incubation with collagenase, which was only incubated for 1 h. The dissociated cellular suspensions were filtered with a 70-μm filter before being pelleted. Cells were either immediately used for various experiments or frozen down in BAM Banker (FujiFilm; CS-02-001). For isolation of normal mouse epidermis, 7-week-old female C57BL/6 J mice were shaved, back skin removed, and underlying fat was scrapped off. The skin was then minced into pieces that were smaller than 1 mm in diameter. Minced samples were placed in 15-mL conical tubes and digested with 10 mL of 0.25% collagenase solution in DKFSM. Samples were incubated at 37 °C with rotation for 2 h. 1 mL of FBS was then added to the cellular suspension. The dissociated cellular suspensions were filtered with a 70-μm filter before being pelleted and immediately used for downstream experiments.

### Flow cytometry/fluorescence-activated cell sorting (FACS)

For FACS or flow experiments, single-cell suspensions of cells were resuspended in a 2% FBS solution and then stained with appropriate antibodies at a 1:100 dilution for 60 min at 4 °C in the dark. Antibodies used were FITC anti-mouse Ly6d (BioLegend; 138606) and APC anti-human/mouse Cd49f (BioLegend; 313616). Before sorting or flow, SytoxBlue (Thermo Fisher; S34857) was used for a viability dye at a 1:1000 dilution. FACS experiments were run on FACSAria II, and flow experiments were run on LSRII instruments. Both instruments used the BD FACSDiva 8.0.1 software for data collection. For scRNA-Seq experiments, cells were only stained with SytoxBlue, and live cells were sorted for downstream processing and experimentation. For EdU experiments, we used the Click-iT Plus EdU Alexa Flour 647 Flow Cytometry Assay Kit as per manufacturer instructions (Invitrogen; C10634). For Apotracker^TM^ experiments, we used the Apotracker^TM^ Green reagent as per manufacturer instructions (BioLegend; 427402). FlowJo 10.6 was used for data analysis.

### scRNA-Seq library preparation and sequencing

As per the directions of the Chromium Single-Cell 3ʹ Reagents Kit v2 (following the CG00052 Rev B. user guide), human or mouse live FACS-sorted cells were washed in PBS containing 0.04% BSA and resuspended at a concentration of ~1000 cell/μL. We aimed to capture 10,000 cells. Each library was sequenced on the Illumina HiSeq 4000 platform to achieve an average of ~30,000 reads per cell.

### scRNA-Seq library processing, quality control, and clustering

FASTQ files were aligned using 10X Genomic Cell Ranger 3.1.0 and aligned to either an indexed hg38 or mm10 for human or mouse tumors, respectively. Standard workflow using Seurat v3 using R 4.2.1 with quality control parameters of 200–5000 gene detection and mitochondrial percentage under 10% were utilized to filter cells. Cells were presented in two-dimensional space using UMAP with the following sample-specific parameters and subset workflow listed below. For human BCC samples, each tumor (BCC19, BCC20, and BCC21) were initially clustered separately from one another to identify tumor epithelial populations using the top 15 PCs with a resolution of 0.3. Based on marker gene expression of epithelial markers (*KRT14*) and tumor-specific markers (*GLI1*), we were able to subset a tumor-specific population for BCC19, BCC20, and BCC21. We used the CopyKAT 1.0.8 program to confirm the discrimination of tumor epithelium from normal tissue as well as examine variations in CNVs of distinct clusters form scRNA-Seq data. We were then able to merge these 3 tumor epithelial populations along with 4 published tumor epithelial populations using the Multiple Dataset Integration and Label Transfer (anchoring) standard workflow within Seurat where the top 15 PCs with a resolution of 0.4 were used^11^. The tumor-specific population for BCC21 was more extensively analyzed using the top 5 PCs with a resolution of 0.3. To analyze both BCC and SCC samples together, the 7 BCC samples were merged with 6 SCC samples using the Multiple Dataset Integration and Label Transfer (anchoring) standard workflow where the top 10 PCs with a resolution of 0.2 were used. To analyze the normal human epidermis, all epithelial cells (subset prior via the epithelial-specific markers of *KRT14* and *KRT1*) from the 5 different datasets were merged using the Multiple Dataset Integration and Label Transfer (anchoring) standard workflow where the top 10 PCs with a resolution of 0.3 were used. For the resistant BCC datasets, we extracted the tumor epithelial populations (as identified through metadata labels) and merged them together for expression analysis^19^. We also specifically extracted and merged the tumor epithelial cells from rBCC6 and rBCC8 for analysis. The primary mouse BCC sample was initially clustered to identify the tumor epithelial cluster using the top 15 PCs with a resolution of 0.3. The tumor epithelial cluster was identified using epithelial markers (*Krt14*) and tumor-specific markers (*Gli1*) and subsequently subsetted and then clustered using the top 10 PCs with a resolution of 0.3. For the mouse drug treatment experiments, the DMSO-treated and Vismodegib-treated tumor samples were first screened for any residual contaminating cells (i.e., non-*Krt14* expressing cells such as fibroblasts) before being merged before using the top 10 PCs with a resolution of 0.2 to cluster. For pancreatic cancer scRNA-Seq analysis, *KRT19*^*+*^ and *AMBP*^+^ ductal cells (along with non-expression of the acinar marker *PRSS1* and endocrine marker CHGB) from 24 different pancreatic cancer and 11 different normal/control samples were extracted and then merged. Initial clustering with the 24 cancer samples and 11 normal samples used the top 15 PCs with a resolution of 0.3. A subset of ductal cells from tumors 1, 6, and 8 were subsequently merged using Multiple Dataset Integration and Label Transfer (anchoring) standard workflow within Seurat. The merged sample was then clustered using the top five PCs with a resolution of 0.2.

### scRNA-seq analysis

Marker genes were found for each individual cluster using the standard Seurat pipeline and parameters such as using log(fold change) >0.25 as a cutoff for performing differential gene expression. COMET 0.1.13 analysis was used to confirm surface markers (https://github.com/MSingerLab/COMETSC). For gene scoring, we used the AddModuleScore function built into Seurat. Gene Ontology analysis was conducted using marker genes for the various clusters, which were submitted to MSigDB collection from the Broad Institute.

### scRNA-seq lineage analysis

For the PCA-based approaches, we simply presented the cells in PCA two-dimensional space using the genes associated with PC1 and PC2. Clusters and variable genes information is pulled from the tumor epithelial population of BCC21 Seurat object and taken as input to construct a pseudo-time trajectory using the semi-supervised Monocle 2 (2.14.0) workflow.

### scATAC-seq library preparation and sequencing

As per the directions of the Chromium Next GEM Single-Cell ATAC Library & Gel Bead Kit v1.1, additional single-cell suspensions of BCC19, BCC20, and BCC21 were FACS-sorted for live cells were subsequently washed in PBS containing 0.04% BSA and resuspended at a concentration of ~1000 cell/μL. We aimed to capture 10,000 cells. Each library was sequenced on the Illumina HiSeq 4000 platform to achieve an average of ~30,000 reads per cell.

### scATAC-seq library processing, quality control, and clustering

For the scATAC-Seq library processing, ‘cellranger-atac count’ function (cellranger-atac, v1.1.0) is used for pre-processing of the 10×-based scATAC-seq data using hg38 human genome. scATAC-Seq analysis was carried out using the R package ArchR (1.0.1). Within ArchR, ‘Arrow files’ are generated for each sample from the fragment file with the parameters of 1000 unique fragments and a transcription-start-site enrichment value of 4. An ‘ArchRproject’ was created for (1) a merged dataset where all arrow files from all three samples (BCC19, BCC20, and BCC21) and (2) BCC21 alone. We used the ‘addIterativeLSI’ function of ArchR to perform dimensionality reduction on each ArchRproject and applied the Harmony 0.1.0 algorithm to mitigate batch effects for the merged ArchRproject. For clustering, the ‘addClusters’ function was used with a resolution of 0.4 for the merged dataset and 0.3 for the BCC21 dataset. Samples were plotted using UMAP with the ‘addUMAP’ followed by the ‘plotEmbedding’ function. To subset cells, the ‘subsetArchRproject’ function is used to isolate cells for subsequent analysis. To identify epithelial cells, which are composed of both tumor epithelial cells and ‘normal’/adjacent epithelial cells from the epidermis, we used the KRT14 GeneScoreMatrix to separate from other cell types such as fibroblasts and immune cells. To distinguish tumor epithelial cells and ‘normal’/adjacent epithelial cells from the epidermis, we used the GLI1 GeneScoreMatrix, which was specific to the tumor. We created pseudo-bulk replicates for each cluster and called peaks using MACS2 2.27.1, identified peaks unique to each cluster, and used ‘getMarkers’ function to obtain the marker list of each cluster. We then added motif annotation to ArchRproject with the function ‘addMotifAnnotations’, which identified enriched motifs associated with differential peaks. We applied the ‘addImputeWeights’ function to impute the weights of marker features and the ‘plotEmbedding’ function to visualize the marker features. We performed pairwise comparisons of peaks and TFs between clusters using the getMarkerFeatures function and plotted an MA or volcano plot using the ‘markerPlot’ function in ArchR. ‘addCoAccessibility’ function is used to examine co-accessibility which returned a loop track that represented the co-accessibility information using the ‘getCoAccessibility’ function. Finally, ‘plotBrowserTrack’ function is used to plot genome browser tracks of peaks and co-accessibility. Later, we defined the trajectory backbone of cell groups or clusters based on BCC state. We then created a trajectory using the ‘addTrajectory’ function and plotted the pseudotime values on UMAP embedding using the ‘plotTrajectory’ function.

### RNAScope

All RNAScope experiments were performed using the Multiplex Fluorescent v2 system (ACD; 323100) as per manufacture protocols. Briefly, human and mouse tumors were fixed with 4% paraformaldehyde overnight at 4 °C, paraffin-embedded, and sectioned at 5 μm. We then followed the formalin-fixed, paraffin-embedded protocol as per manufacturer instructions. The following human probes were used: Hs-*LY6D* (ACD; 484681), Hs-*LYPD3* (ACD; 843431-C3), Hs-*TACSTD2* (ACD; 405471-C4), and Hs-*GLI1* (ACD; 310991-C2). The mouse probe Mm-*Ly6d* (ACD; 532071) was used. After completing the RNAScope protocol, sections were immediately blocked, stained with K14 antibody (1:500; BioLegend SIG-3476-100) for BCC samples or with K19 antibody (1:500; BioLegend 628502) for pancreatic samples, and followed by staining with the anti-chicken Alexa488 secondary antibody (1:500; Invitrogen; A-11039) or anti-mouse Alexa488 secondary antibody (1:500; Invitrogen; A-28175). Nuclei visualization was with Hoechst 33342 (1:1000; ThermoFisher; 62249). Imaging was conducted using a Lecia SP8 with the Lecia LAS X 3.7.6 software. Images were analyzed in FIJI 2.

### Organoid model

Organoid model was based on mouse epidermal and HF organoid culture^29^. Briefly, single-cell suspensions of bulk tumor or post-FACS-sorted mouse tumor epithelial cells were plated at a density from 2500 to 10,000 cells per 10 μL BME (Trevigen; 2432-005-01) drop in a 6-well plate, with 8 drops per well. Cells were cultured in EEM as previously described^29^. Briefly, the contents include: Advanced DMEM/F12 (Gibco; 12491015) supplemented with penicillin/streptomycin (100 U/L; Gibco; 15140122), Hepes, (10 mM; Gibco; 15630080), GlutaMAX (1×; Gibco; 35050061), 1× B27 supplement (50× stock from Gibco; 17504044), N-Acetylcysteine-1 (1 mM; Sigma Aldrich; A9165-5G), Noggin-conditioned medium (5%, derived from HEK293-mNoggin-Fc cells, provided by Hans Clevers), R-spondin1-conditioned medium (5%, derived from HEK293-HA-R-Spondin1-Fc cells; R&D Systems; 3710-001-01), acidic FGF1 (100 ng/mL; PeproTech; 100–17 A), Heparin (0.2%; Stemcell Technologies; 7980), Forskolin (10 ng/mL; Tocris; 1099), Rho kinase inhibitor (Y-27632; 10 μM; Stemcell Technologies; 72302), and 1× Primocin (500× stock; InvivoGen; ant-pm-1). We also found that adding TGF-β inhibitor SD208 (1 μM; Tocris; 3269) enhanced growth. The media was replenished every 3 days. Organoids were grown for 14 days and were subsequently counted or dissociated into single cells for downstream applications. Single-cell dissociation was achieved as previously described^29^. Briefly, the BME drop was disrupted, collected in 15-mL conical tube, and then spun down. The media was removed, 2 mL of TrypLE (Gibco; 12604013) was added to the pelleted organoids, and then incubated for 20 min at 37 °C. After 20 min, cells were dissociated using a P1000 by pipetting up and down more than 10 times. The TrypLE was inactivated by the addition of 1 mL of FBS, washed with 1× PBS, spun down, and then used for downstream applications. For organoid drug treatments, unsorted single-cell suspensions of mouse tumor were plated as described above. For acute drug treatments, organoids were first plated for 7 days, and then the following drugs were added for 48 h before collection: Vismodegib (1 μM; Selleckchem; S1082) and CCG (1 μM; Cayman Chemical; 15075). Media was replaced every 3 days. For long-term drug treatments, at the time of plating, the following drugs were added: Vismodegib (1 μM or 10 μM; Selleckchem; S1082), CCG (1 μM; Cayman Chemical; 15075), and Runx1 inhibitor Ro 5–335 (1 μM or 10 μM; Tocris; 4694). The organoids were then cultured for 14 days with media and drugs replaced every 3 days. For organoid antibody treatments, cells were plated and then immediately treated with anti-Ly6d (10 μg/mL; Proteintech; 17361-1-AP) or anti-IgG (10 μg/mL; Millipore Sigma; 12-371) for 7 days before images were taken.

### CRISPR in ASZs

ASZ_001 cells (known as ASZs) were cultured as previously described in M154CF (ThermoFisher; M154CF500) with 2% chelated FBS, 1% penicillin/streptomycin (Gibco; 15140122), and 0.05 mM CaCl_2_^30^. 250,000 cells were nucleofected with gRNAs for Foxn3 or Runx1 (Synthego). Briefly, RNPs were generated by combining 0.24nmol gRNAs with 12.9 μg Cas9 proteins (TrueCut Cas9 Protein v2; ThermoFisher; A36498) and incubated at room temperature for 10 min before being placed on ice. Nucleofection was carried out with the Amaxa Cell Line Nucleofector Kit T (Lonza; VCA-1002) in conjunction with the Amaxa Nucleofector II, following the manufacturer’s protocols using the T-029 program. Pure knockout clones were isolated through serial dilution of the nucleofected cells. Clones were genotyped with primers designed to span the cut sights. Once clones were isolated, their deletions were confirmed by Sanger sequencing and ICE analysis (Synthego). ASZ_001 cells were derived from mouse basal cell carcinoma cells and previously authenticated^30^. In our experiments, cells were monitored by their morphology, sequencing, and utilization of mouse-specific primers. No mycoplasma was detected through regular testing.

sgRNA sequences:

Runx1-1: AGGAGTACCTTGAAAGCGAT

Runx1-2: GGCTCGTGCTGGCATCTACG

Runx1-3: TAGCGAGATTCAACGACCTC

Foxn3-1: GCAGAAACTGCTAACCAGAC

Foxn3-2: ACGGAGCCCTCCTGCAAGGT

Foxn3-3: GTGTTCTCTTCCAGCACATC

Genotyping primers:

Runx1-F: GCCCGATCCAAGGTTCTAACT

Runx1-R: CCTGTCGCCGTCTAGTAGGA

Foxn3-F: CAACTGTCACACCATCCCGA

Foxn3-R: GATGCTTTTTGCCTTGGCGA

### Mouse drug treatments

For mouse drug treatments, we passaged 1 million cells from primary tumors into NOD SCID mice (2 mice per primary tumor). We then injected either DMSO or Vismodegib (Selleckchem; S1082) with a dose of 100 mg/kg body weight every day for 2 weeks. Mice tumor size was measured with calipers during and then after the last treatment to monitor growth.

### Gene expression

RNA was isolated using TRIzol Reagent (Invitrogen; 15596026) as per the manufacturer’s instructions. For gene expression analysis in organoids, Brilliant II SYBR Green QRT-PCR 1-Step Master Mix (Agilent; 600825) was used for reverse transcription and subsequent quantitative PCR. Each reaction was performed in triplicate and values were normalized to Gapdh. The MxPro software was used for data analysis.

Primers used were:

Ly6d-F: CAAAACCGTCACCTCAGTGGA

Ly6d-R: GTGACCTCGGAACCACTGCT

Gli1-F: CCAAGCCAACTTTATGTCAGGG

Gli1-R: AGCCCGCTTCTTTGTTAATTTGA

Gapdh-F: AATGAATACGGCTACAGCAACAGGGTG

Gapdh-R: AATTGTGAGGGAGATGCTCAGTGTTGGG

For gene expression analysis in ASZs, RNA was isolated from adherent cells using the RNeasy Plus Mini Kit (Qiagen; 74134) according to the manufacturer’s instructions. Quantitative real-time PCR was performed using the TaqMan assay (ThermoFisher; 4392938) and normalized to Gapdh. The following probes were used: Gapdh-FAM Mm99999915_g1 (ThermoFisher; 4331182), Gli1-FAM Mm00494654_m1 (ThermoFisher; 4331182), Tacstd2-FAM Mm00498401_s1 (ThermoFisher; 4331182), and Lypd3-FAM Mm00519321_m1 (ThermoFisher; 4331182).

### Pancreatic analysis using the browser

To examine the expression levels of LY6D in various tumor types as well as look at survival data, we utilized the GEPIA web interface (http://gepia.cancer-pku.cn/).

### Reporting summary

Further information on research design is available in the Nature Portfolio Reporting Summary linked to this article.

## Supplementary information

Supplementary Information

Description of Additional Supplementary Files

Supplementary Data 1

Supplementary Data 2

Supplementary Data 3

Supplementary Data 4

Supplementary Data 5

Supplementary Data 6

Supplementary Data 7

Supplementary Data 8

Supplementary Data 9

Supplementary Data 10

Reporting Summary

## Data Availability

The human-specific sequencing data generated from this study have been deposited in the dbGAP database under the accession code phs003103.v1.p1 (http://www.ncbi.nlm.nih.gov/projects/gap/cgi-bin/study.cgi?study_id=phs003103.v1.p1). Patient data lies under restricted access on dbGAP but will be perpetually available for general use through dbGAP for those with appropriate institutional human subjects approval (above database link also contains instructions for access). The mouse-specific sequencing data generated from this study have been deposited in the NCBI Gene Expression Omnibus under the SuperSeries accession code: GSE186184. The naive human BCC scRNA-Seq publicly available data used in this study are available in the NCBI Gene Expression Omnibus under the accession code: GSE141526^11^. The resistant human BCC scRNA-Seq publicly available data used in this study are available in the NCBI Gene Expression Omnibus under the accession code: GSE123814^19^. The human SCC scRNA-Seq publicly available data used in this study are available in the NCBI Gene Expression Omnibus under the accession code: GSE144240^17^. The remaining data are available within the Article, Supplementary Information, or Source Data file. Source data are provided with this paper.

## Source data

Source Data
