# Supplementary material for: LY6D marks pre-existing resistant basosquamous tumor subpopulations
Source: Nat Commun. 2022 Dec 6;13:7520. doi: 10.1038/s41467-022-35020-y (PMC9726704; doi:10.1038/s41467-022-35020-y)
Supplement: Supplementary file 1 — Supplementary Information [file 41467_2022_35020_MOESM1_ESM.pdf]

Supplementary Information:

LY6D marks pre-existing resistant basosquamous tumor subpopulations

Daniel Haensel[1], Sadhana Gaddam[1], Nancy Y. Li[1], Fernanda Gonzalez[1], Tiffany Patel[1], Jeffrey M. Cloutier[2], Kavita Y. Sarin[3], Jean Y. Tang[3], Kerri E. Rieger[2,3], Sumaira Z. Aasi[3], and Anthony E. Oro[1,4]

[1]Program in Epithelial Biology, Stanford University School of Medicine, Stanford CA, United States of America
[2]Department of Pathology, Stanford University School of Medicine, Stanford CA, United States of America
[3]Department of Dermatology, Stanford University School of Medicine, Stanford CA, United States of America
[4]Correspondence to: Anthony E. Oro MD/PhD, Program in Epithelial Biology, Stanford University School of Medicine, Stanford CA, United States of America, Phone: +1 650-723-7843, Fax: +1 650-723-8762, E-mail: oro@stanford.edu

SUPPLEMENTARY FIGURE 1

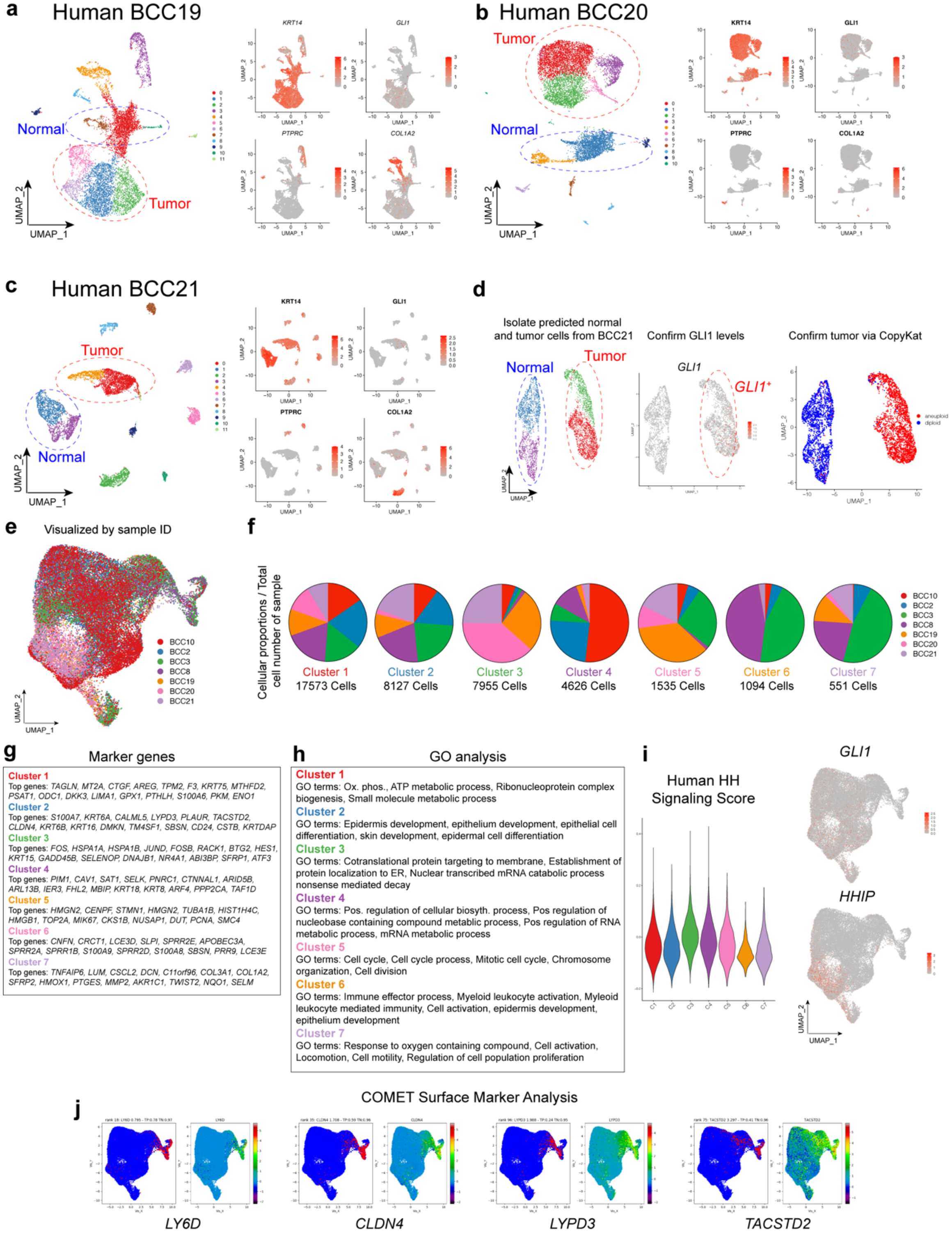

Supplementary Figure 1: Overview of human BCC datasets

a. UMAP plot of scRNA-Seq for human BCC19 and feature plots with expression for major cell types (*KRT14*, *GLI1*, *PTPRC*, and *COL1A2*). Tumor epithelial cells (*KRT14*$^{+}$*GLI1*$^{+}$) are circled.
b. UMAP plot of scRNA-Seq for human BCC20 and feature plots with expression for major cell types (*KRT14*, *GLI1*, *PTPRC*, and *COL1A2*). Tumor epithelial cells (*KRT14*$^{+}$*GLI1*$^{+}$) are circled.
c. UMAP plot of scRNA-Seq for human BCC21 and feature plots with expression for major cell types (*KRT14*, *GLI1*, *PTPRC*, and *COL1A2*). Tumor epithelial cells (*KRT14*$^{+}$*GLI1*$^{+}$) are circled.
d. UMAP plot of human BCC21 tumor and normal epithelium subset from Supplementary Fig. 1c with a feature plot for *GLI1*. CopyKAT analysis projected onto the same UMAP.
e. UMAP plot of scRNA-Seq data from seven merged human BCC tumor epithelial samples shown by the different tumor identities from Fig. 1b.
f. Proportions of the seven different tumors across all the different clusters from Fig. 1b.
g. Top marker genes for the seven different clusters in the human BCC dataset in Fig. 1b.
h. GO analysis of the marker genes from the seven different clusters in the human BCC dataset in Fig. 1b.
i. Gene scoring for HH signaling and feature plots for *GLI1* and *HHIP* for the merged human BCC dataset showing reduced score for Merged-Hu-C6.
j. Plots from Comet verifying LY6D, CLDN4, LYPD3, and TACSTD2 surface markers.

SUPPLEMENTARY FIGURE 2

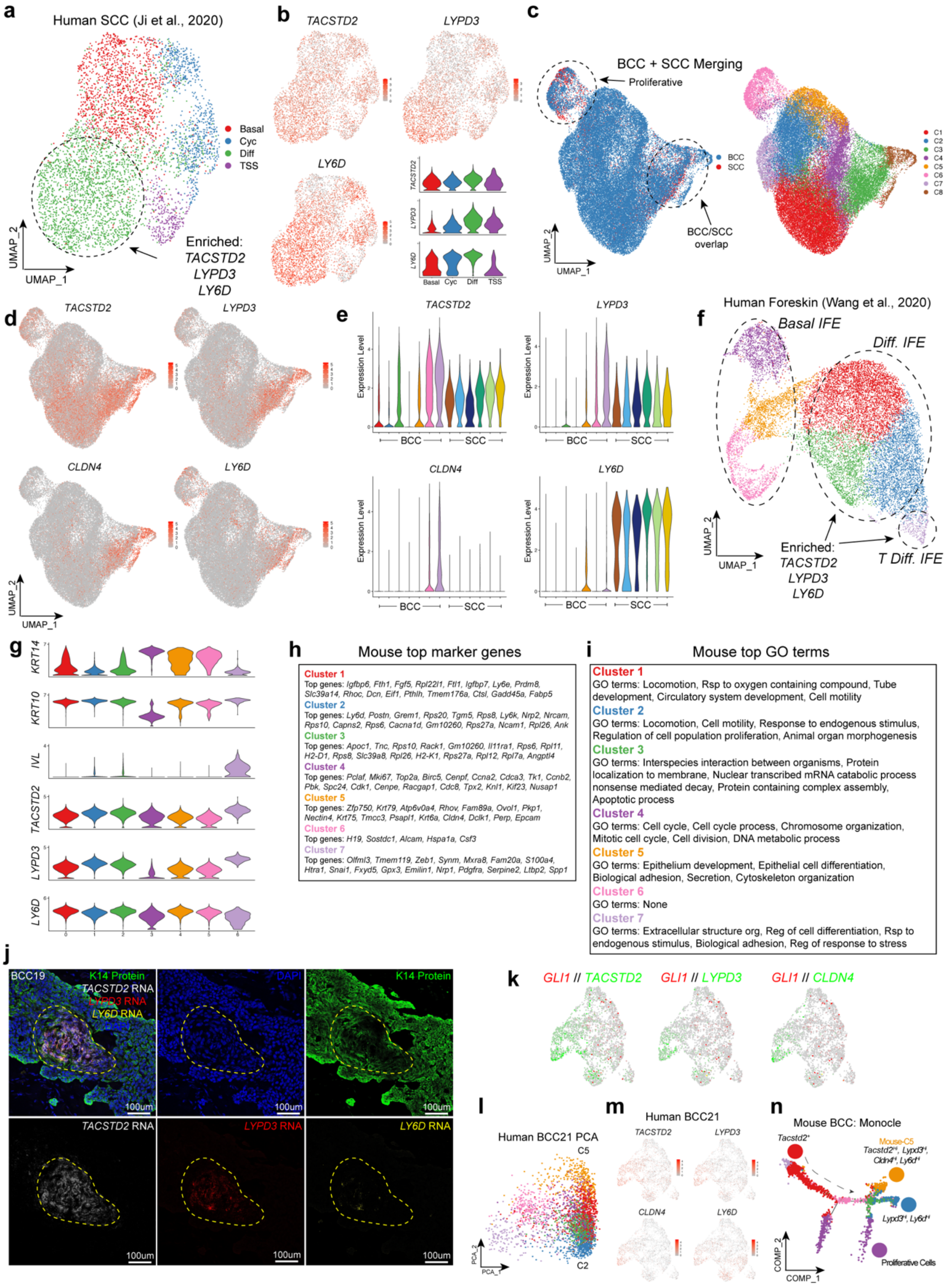

Supplementary Figure 2: Additional scRNA-Seq analysis

a. UMAP plot of scRNA-Seq data from human SCC, where clusters are labeled by cell states indicated by *Ji et al., 2020*.
b. Feature plots and violin plot of the different basosquamous markers (*TACSTD2*, *LYPD3*, and *LY6D*) for the human SCC dataset in Supplementary Fig. 2a.
c. Merged human BCC and SCC datasets colored by tumor type identity (left) or clustering (right). Tumor epithelial cells were isolated from each sample and subsequently merged via Multiple Dataset Integration and Label Transfer (anchoring).
d. Feature plots of the different basosquamous markers (*TACSTD2*, *LYPD3*, *CLDN4*, and *LY6D*) for the merged human BCC and SCC sample in Supplementary Fig. 2c.
e. Violin plots for the various basosquamous markers (*TACSTD2*, *LYPD3*, *CLDN4*, and *LY6D*) for each BCC and SCC tumor sample that was merged in Supplementary Fig. 2c.
f. UMAP plot of scRNA-Seq from human foreskin epidermis.
g. Violin plot for various lineage markers (KRT14, KRT10, and IVL) and basosquamous markers (*TACSTD2*, *LYPD3*, and *LY6D*) from the human foreskin epidermis in Supplementary Fig. 2f.
h. Top marker genes for the different clusters in the mouse BCC dataset in Fig. 1h
i. GO analysis of the marker genes for the different clusters in the mouse BCC dataset in Fig. 1h.
j. Spatial localization of *TACSTD2* (white), *LYPD3* (red), and *LY6D* (yellow) transcripts in human BCC19, which are co-stained with K14 protein (green). n = 3. Scale bar is 100 um.
k. Feature plot human BCC21 showing dual expression of *GLI1* and *TACSTD2, LYPD3, or CLDN4.*
l. PCA projection of human BCC21 tumor from Fig. 2b.
m. Feature plot of the different basosquamous markers (*TACSTD2*, *LYPD3*, *CLDN4*, and *LY6D*) from human BCC21 tumor from Fig. 2b.
n. Monocle projection of mouse BCC tumor sample with the same cluster information from Fig. 1h. Arrow directions indicate a transition from a *Tacstd2*$^{+}$ to Mouse-C5, which is *Ly6d*^{Hi}.

SUPPLEMENTARY FIGURE 3

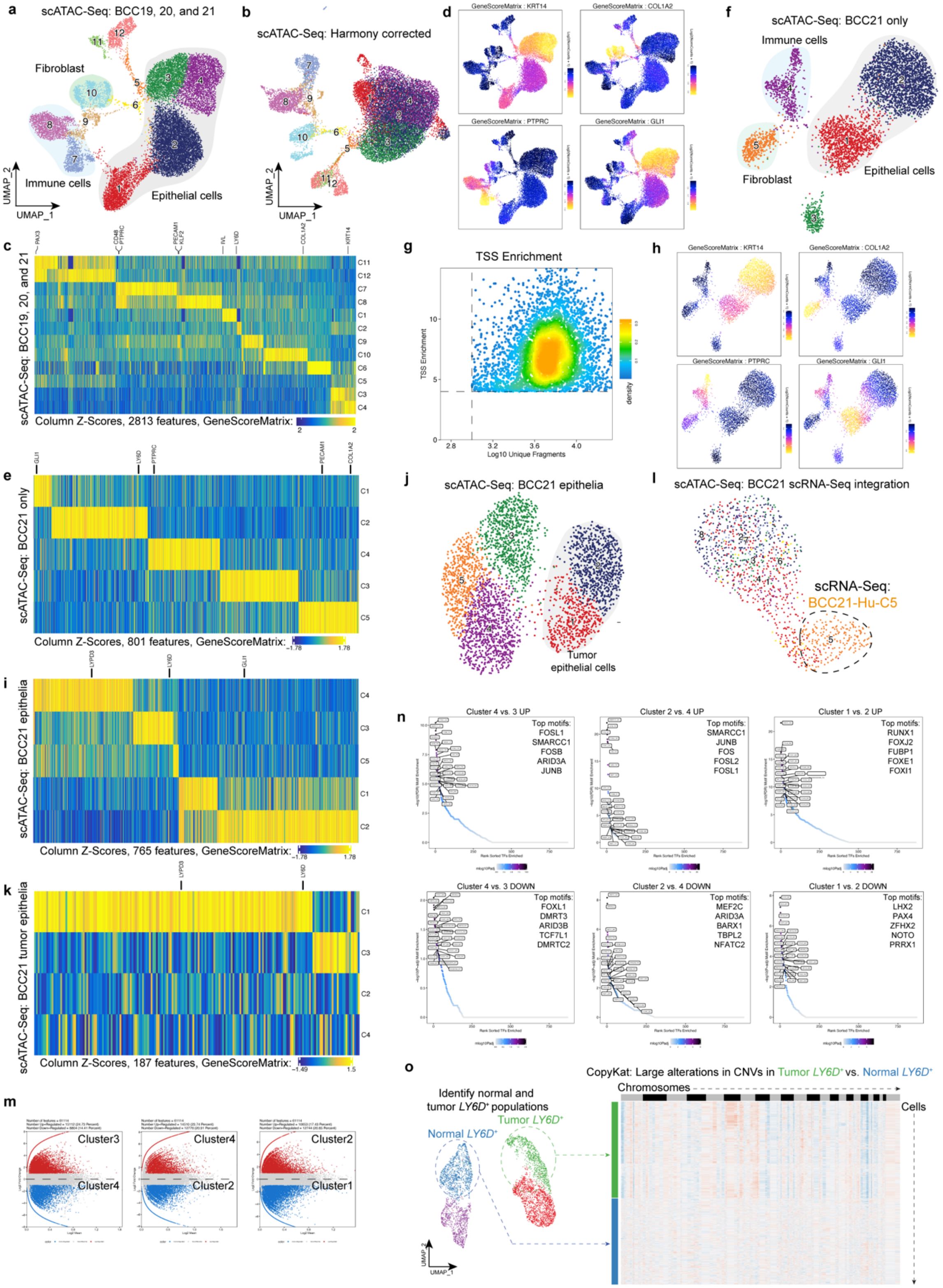

Supplementary Figure 3: Additional lineage analysis of scRNA-Seq data and overview of scATAC-Seq analysis

a. UMAP plot of scATAC-Seq data from 3 merged human BCC tumor samples identified the major cell types from Fig. 2d.
b. UMAP plot of Harmony corrected scATAC-Seq data from 3 merged human BCC tumor samples by identifying the major cell types from Fig. 2d.
c. Heatmap of all the major features associated with each cluster of the merged scATAC-Seq sample shown in Supplementary Fig. 3d.
d. Feature plot of the GeneScoreMatrix for KRT14, GLI1, PTPRC, and COL1A2 for the merged scATAC-Seq sample shown in Supplementary Fig. 3d.
e. Heatmap of all the major features associated with each cluster of human BCC21 scATAC-Seq sample.
f. UMAP plot of the scATAC-Seq sample for human BCC21 with identification of the major cell types.
g. TSS enrichment plot for human BCC21 sample.
h. Feature plot of the GeneScoreMatrix for KRT14, GLI1, PTPRC, and COL1A2 for human BCC21 sample in Supplementary Fig. 3i.
i. Heatmap of all the major features associated with all epithelial populations (tumor and normal) of human BCC21 scATAC-Seq sample.
j. UMAP plot of the scATAC-Seq data for human BCC21 epithelial populations (tumor and normal).
k. Heatmap of all the major features associated with each cluster of the tumor epithelial populations of human BCC21.
l. Integration of the human BCC21 tumor scRNA-Seq with the scATAC-Seq data
m. Differential marker peak analysis between clusters in human BCC21 tumor scATAC-Seq analysis.
n. Motif enrichment analysis between different clusters in the human BCC21 tumor scATAC-Seq analysis. Cutoffs used were a FDR < 0.1 and Log2FC <-0.5.
o. Heatmap of CNV analysis of the *LY6D*$^{+}$ populations from the normal and tumor epithelium.

SUPPLEMENTARY FIGURE 4

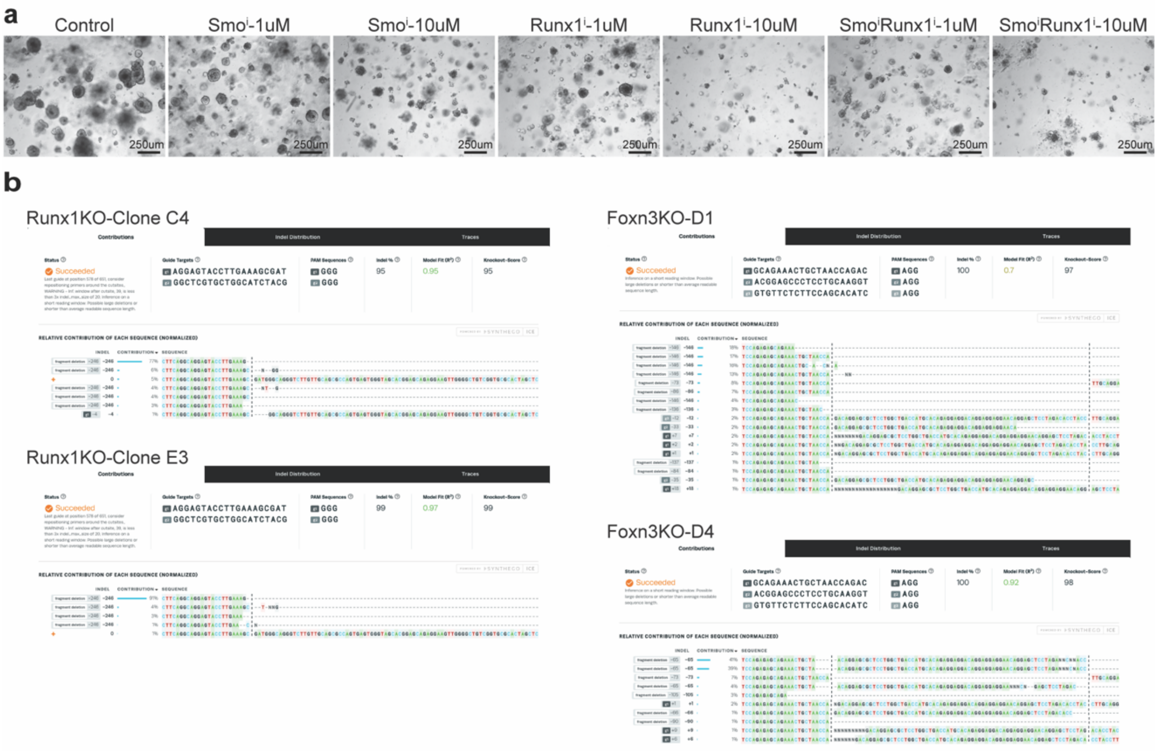

Supplementary Figure 4: Functional experiments with Runx1 and Foxn3

a. Brightfield images of organoids from mouse BCC tumors that were treated with SMO^i, $Runx1^i$, or SMO^i with $Runx1^i$. Inhibitor treatments were at 1uM or 10uM as indicated. n = 3.
b. ICE analysis for Runx1KO and Foxn3KO clones. Runx1KO clone C4 is a 246bp deletion with a Knockout Score of 95, Runx1KO clone E3 is a 246bp deletion with a Knockout Score of 99, Foxn3KO clone D1 is a 146bp deletion with a Knockout Score of 97, and Foxn3KO clone D4 is a 65bp deletion with a Knockout Score of 98.

Length of each scale bar is noted in figure.

SUPPLEMENTARY FIGURE 5

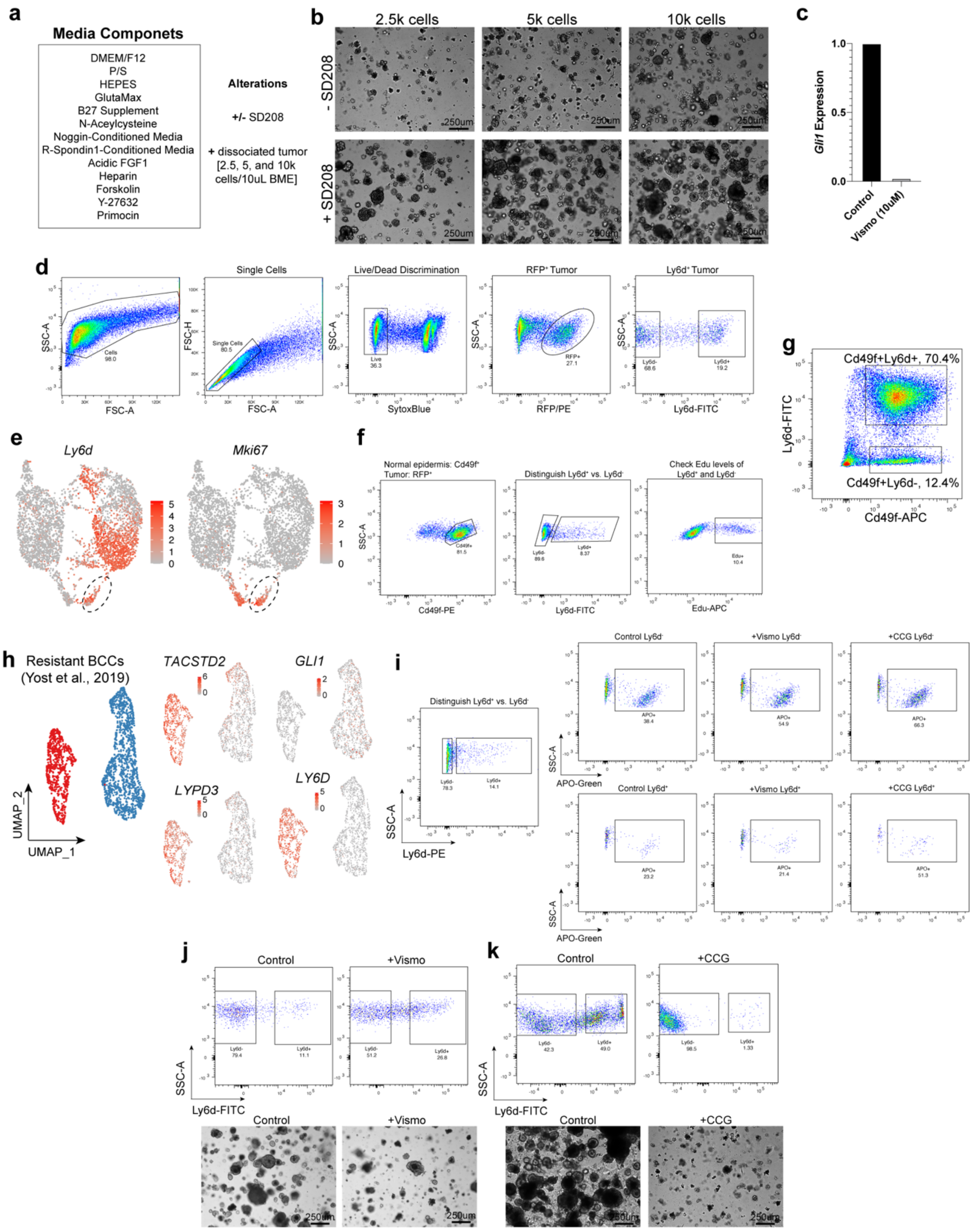

Supplementary Figure 5: Organoids and drug treatments

a. Overview of the media components and various alterations for organoid growing media.
b. Brightfield images of organoids grown at different concentrations with and without the addition of SD208. n = 1.
c. The expression level of *Gli1* in organoids after treatment with SMO^{i}.
d. Flow cytometry schematic for organoids used to assess levels of Ly6d after dissociation. Example panels are relevant to Fig. 2k-m, 3f, 5c, and d.
e. Feature plots for expression levels of *Ly6d* and *Mki67* in mouse BCC scRNA-Seq sample Fig. 1h.
f. Flow cytometry schematic for assessing Edu% in organoids. Example panel is relevant to Fig. 3f.
g. Flow cytometry plot for how $Ly6d^{+}$ cells were identified in normal mouse epidermis. Example panel is relevant to Fig. 2k and l.
h. UMAP plot for two resistant human BCC samples and feature plots for *LY6D, LYPD3, TACSTD2,* and *GLI1.*
i. Flow cytometry example assessing the levels of Apotracker Green after SMO^{i} and CCG treatments in either $Ly6d^{-}$ or $Ly6d^{+}$ cells. Example panels are relevant to Fig. 5c.
j. Flow cytometry example assessing Ly6d levels after SMO^{i} and corresponding brightfield images of organoids. Example panels are relevant to Fig. 5d.
k. Flow cytometry example assessing Ly6d levels after CCG and corresponding brightfield images of organoids. Example panels are relevant to Fig. 5d.

Length of each scale bar is noted in figure.

SUPPLEMENTARY FIGURE 6

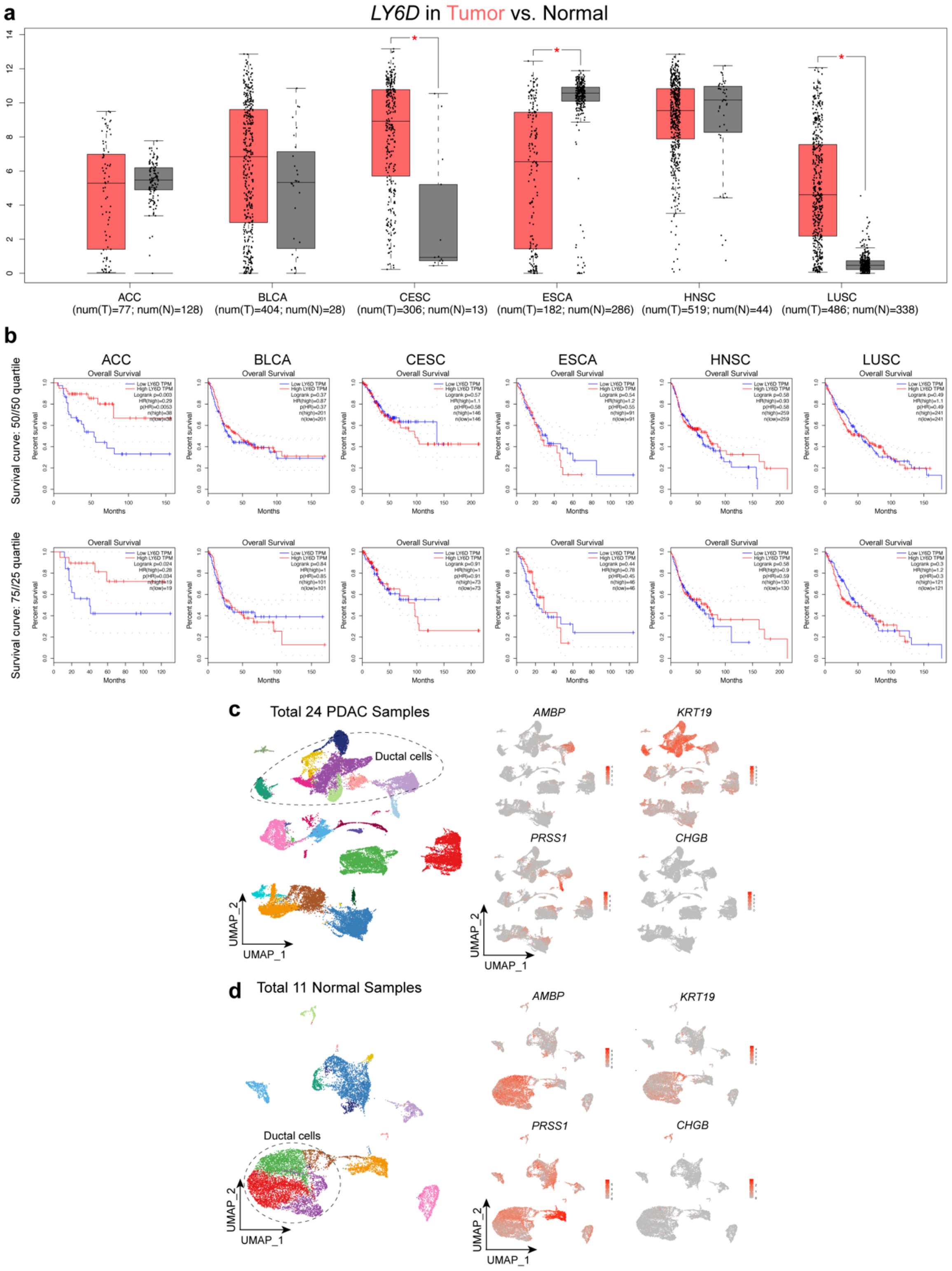

Supplementary Figure 6:

a. Bulk gene expression profile of *LY6D* between ACC (77 tumor and 128 normal), BLCA (404 tumor and 28 normal), CESC (306 tumor and 13 normal), ESCA (182 tumor and 286 normal), HNSC (519 tumor and 44 normal), and LUSC (486 tumor and 338 normal) cancers with corresponding control/normal tissue. Box and whisker boundaries indicate the 25th and 75th percentile, limits indicate minima and maxima values, and center value indicating the mean.
b. Survival curves between for ACC (50//50 quartile: 38 patients in high and low *LY6D* groups; 75//25 quartile: 19 patients in high and low *LY6D* groups), BLCA (50//50 quartile: 201 patients in high and low *LY6D* groups; 75//25 quartile: 101 patients in high and low *LY6D* groups), CESC (50//50 quartile: 146 patients in high and low *LY6D* groups; 75//25 quartile: 73 patients in high and low *LY6D* groups), ESCA (50//50 quartile: 91 patients in high and low *LY6D* groups; 75//25 quartile: 46 patients in high and low *LY6D* groups), HNSC (50//50 quartile: 259 patients in high and low *LY6D* groups; 75//25 quartile: 130 patients in high and low *LY6D* groups), and LUSC (50//50 quartile: 241 patients in high and low *LY6D* groups; 75//25 quartile: 121 patients in high and low *LY6D* groups) cancers with patients that have high and low levels of *LY6D.* Two different quartile cutoffs are shown.
c. UMAP plot of scRNA-Seq for the 24 PDAC samples and feature plots with expression for major cell types (*AMBP*, *KRT19*, *PRSS1*, and *CHGB*) were used to identify ductal cells.
d. UMAP plot of scRNA-Seq for the 11 normal pancreas samples and feature plots with expression for major cell types (*AMBP*, *KRT19*, *PRSS1*, and *CHGB*) were used to identify ductal cells.

For a, *p*-values were calculated using a one-way ANOVA with the '*' indicating a $p<0.01$
