# Supplementary material for: LY6D marks pre-existing resistant basosquamous tumor subpopulations
Source: Nat Commun. 2022 Dec 6;13:7520. doi: 10.1038/s41467-022-35020-y (PMC9726704; doi:10.1038/s41467-022-35020-y)
Supplement: Supplementary file 2 — Description of Additional Supplementary Files [file 41467_2022_35020_MOESM2_ESM.pdf]

Description of Additional Supplementary Files

File Name: Supplementary Data 1
Description: Marker genes form the scRNA-Seq clusters of the 7 merged BCCs

File Name: Supplementary Data 2
Description: Marker genes from the scRNA-Seq clusters of mouse BCC

File Name: Supplementary Data 3
Description: Gene overlap between Merge-Hu-C6 and Mouse-C5

File Name: Supplementary Data 4
Description: Marker genes from the scRNA-Seq clusters of human BCC21

File Name: Supplementary Data 5
Description: Marker features from the scATAC-Seq clusters of the 3 human BCCs

File Name: Supplementary Data 6
Description: Marker features from the scATAC-Seq clusters of human BCC21

File Name: Supplementary Data 7
Description: Marker features from the scATAC-Seq clusters of the epithelial populations from human BCC21

File Name: Supplementary Data 8
Description: Marker features from the scATAC-Seq clusters of the tumor epithelial populations from human BCC21

File Name: Supplementary Data 9
Description: Differential marker features between normal and tumor epithelial populations

File Name: Supplementary Data 10
Description: Marker genes from the scRNA-Seq clusters of control and SMOi treated mouse tumors
