# Supplementary material for: LY6D marks pre-existing resistant basosquamous tumor subpopulations
Source: Nat Commun. 2022 Dec 6;13:7520. doi: 10.1038/s41467-022-35020-y (PMC9726704; doi:10.1038/s41467-022-35020-y)
Supplement: Supplementary file 13 — Reporting Summary [file 41467_2022_35020_MOESM13_ESM.pdf]

nature portfolio

Corresponding author(s): Anhtony Oro

Last updated by author(s): Nov 7, 2022

Reporting Summary

Nature Portfolio wishes to improve the reproducibility of the work that we publish. This form provides structure for consistency and transparency in reporting. For further information on Nature Portfolio policies, see our Editorial Policies and the Editorial Policy Checklist.

Statistics

For all statistical analyses, confirm that the following items are present in the figure legend, table legend, main text, or Methods section.

n/a	Confirmed	
☐	☒	The exact sample size (*n*) for each experimental group/condition, given as a discrete number and unit of measurement
☐	☒	A statement on whether measurements were taken from distinct samples or whether the same sample was measured repeatedly
☐	☒	The statistical test(s) used AND whether they are one- or two-sided<br>*Only common tests should be described solely by name; describe more complex techniques in the Methods section.*
☒	☐	A description of all covariates tested
☐	☒	A description of any assumptions or corrections, such as tests of normality and adjustment for multiple comparisons
☐	☒	A full description of the statistical parameters including central tendency (e.g. means) or other basic estimates (e.g. regression coefficient) AND variation (e.g. standard deviation) or associated estimates of uncertainty (e.g. confidence intervals)
☐	☒	For null hypothesis testing, the test statistic (e.g. *F*, *t*, *r*) with confidence intervals, effect sizes, degrees of freedom and *P* value noted<br>*Give P values as exact values whenever suitable.*
☒	☐	For Bayesian analysis, information on the choice of priors and Markov chain Monte Carlo settings
☒	☐	For hierarchical and complex designs, identification of the appropriate level for tests and full reporting of outcomes
☒	☐	Estimates of effect sizes (e.g. Cohen's *d*, Pearson's *r*), indicating how they were calculated

Our web collection on statistics for biologists contains articles on many of the points above.

Software and code

Policy information about availability of computer code

Data collection	The various commercial/open source software was used for data collection: BD FACSDiva v8.0.1, Lecia LAS X v3.7.6, Illumina HiSeq and NextSeq System Suite, and MxPro v4.10
Data analysis	The various commercial/open source software was used for data analysis: Seurat v3, CopyKat v1.0.8, COMET v 0.1.13, FIJI 2, 10x Genomics Cell Ranger v3.1.0, R v4.2.1, Graphpad Prism 8, Monocle 2 v2.14.0, ArchR v1.0.1, FlowJo v10.6, GEPIA, MACS2 v 2.27.1, and Harmony v0.1.0

For manuscripts utilizing custom algorithms or software that are central to the research but not yet described in published literature, software must be made available to editors and reviewers. We strongly encourage code deposition in a community repository (e.g. GitHub). See the Nature Portfolio guidelines for submitting code & software for further information.

Data

Policy information about availability of data

All manuscripts must include a data availability statement. This statement should provide the following information, where applicable:

- Accession codes, unique identifiers, or web links for publicly available datasets
- A description of any restrictions on data availability
- For clinical datasets or third party data, please ensure that the statement adheres to our policy

The human specific sequencing data generated from this study have been deposited in the dbGAP database and can be accessed with the hyperlink http://www.ncbi.nlm.nih.gov/projects/gap/cgi-bin/study.cgi?study_id=phs003103.v1.p1. The mouse specific sequencing data generated form this study have been

deposited in the NCBI Gene Expression Omnibus under the SuperSeries accession code: GSE186184. The naïve human BCC scRNA-Seq publicly available data used in this study are available in the NCBI Gene Expression Omnibus under the accession code: GSE141526. The resistant human BCC scRNA-Seq publicly available data used in this study are available in the NCBI Gene Expression Omnibus under the accession code: GSE123814. The human SCC scRNA-Seq publicly available data used in this study are available in the NCBI Gene Expression Omnibus under the accession code: GSE144240.

Human research participants

Policy information about studies involving human research participants and Sex and Gender in Research.

Reporting on sex and gender	All patient samples have been completely de-identified as per IRB protocols and we do not have this information.
Population characteristics	All patient samples have been completely de-identified as per IRB protocols and we do not have this information.
Recruitment	Patients are asked by physician if they would like to participate at time. There is no obvious self-selection bias or other biases that are present.
Ethics oversight	All patient samples were obtained through written informed consent and subsequently de-identified. All protocols for sample acquisition and usage are in accordance with the reviewed protocol by the Stanford University Institutional Review Board, protocol #18325 (Stanford, CA).

Note that full information on the approval of the study protocol must also be provided in the manuscript.

Field-specific reporting

Please select the one below that is the best fit for your research. If you are not sure, read the appropriate sections before making your selection.

☒ Life sciences ☐ Behavioural & social sciences ☐ Ecological, evolutionary & environmental sciences

For a reference copy of the document with all sections, see nature.com/documents/nr-reporting-summary-flat.pdf

Life sciences study design

All studies must disclose on these points even when the disclosure is negative.

Sample size	For all experiments requiring statistical analysis, we aimed to have a minimum of n = 3 biological replicates. We noted exact sizes either within the methods section or the figure legends. Sample size selection was based on similar types of experiments in the literature.
Data exclusions	No data was excluded from analysis.
Replication	All experiments were repeated at a minimum of 2 times will all replication attempts being successful.
Randomization	For all relevant studies/experiments, the same homogeneous sample was randomly split into either a treatment or control groups.
Blinding	Investigators were blind to primary human samples that were acquired for sequencing experiments as they were de-identified. For the various experimental/treatment studies, investigator was not blinded to group allocation because the same investigator conducted data collection and data analysis.

Reporting for specific materials, systems and methods

We require information from authors about some types of materials, experimental systems and methods used in many studies. Here, indicate whether each material, system or method listed is relevant to your study. If you are not sure if a list item applies to your research, read the appropriate section before selecting a response.

Materials & experimental systems

n/a	Involved in the study	
☐	☒	Antibodies
☐	☒	Eukaryotic cell lines
☒	☐	Palaeontology and archaeology
☐	☒	Animals and other organisms
☒	☐	Clinical data
☒	☐	Dual use research of concern

Methods

n/a	Involved in the study	
☒	☐	ChIP-seq
☐	☒	Flow cytometry
☒	☐	MRI-based neuroimaging

Antibodies

Antibodies used	(1) FITC anti-Ly6d (BioLegend; 138606; Clone 49-H4), dilution 1:100<br>(2) APC anti-Cd49f (BioLegend; 313616; Clone GoH3), dilution 1:100<br>(3) anti-K14 (BioLegend; SIG-3476-100; Clone Poly9060), dilution 1:500<br>(4) anti-K19 (BioLegend; 628502; Clone A53-B/A2), dilution 1:500<br>(5) anti-Ly6d (Proteintech; 17361-1-AP), used at 10 μg/mL<br>(6) anti-Chicken Alexa488 (Initrogen; A-11039), dilution 1:500<br>(7) anti-Mouse Alexa488 (Invitrogen; A28175), dilution 1:500<br>(8) anti-IgG (Millipore Sigmal 12-371), used at 10 μg/mL
Validation	(1) Antibody has been validated by manufacturer and has been used in mouse setting previous publications: PMID: 30314756.<br><br>(2) Antibody has been validated by manufacturer and has been used in mouse setting in previous publications: PMID: 33025905<br><br>(3) Exact antibody has been discontinued by manufacturer. The regulatory status has changed from IVD to RUO but the clone remains exactly the same. As such, the catalog number has changed to 906004. Antibody has been validated by manufacturer and has been used in both human and mouse settings in previous publications: PMID: 32521267.<br><br>(4) Antibody has been validated by manufacturer and has been used in human setting in previous publication: PMID: 31763993.<br><br>(5) Antibody has been validated by manufacturer and has been used in mouse setting in previous publications: PMID: 33025905 and PMID: 33033234.<br><br>(6) Antibody has been validated by manufacturer and has been used in both human and mouse settings in previous publications: PMID: 28459457 and PMID: 31868583.<br><br>(7) Antibody has been validated by manufacturer and has been used in both human and mouse settings in previous publications: PMID: 32923629 and PMID: 31794382<br><br>(8) Antibody has been validated by manufacturer and has been used in mouse setting in previous publication: PMID: 29026085

Eukaryotic cell lines

Policy information about cell lines and Sex and Gender in Research

Cell line source(s)	The mouse basal cell carcinoma cell line ASZ001 was generated by Epstein group (So et al., Experimental Dermatology 2006)
Authentication	ASZ001 were authenticated previously by the Epstein group (So et al., Experimental Dermatology 2006). In our experiments, ASZ001 cells are monitored by their morphology, sequencing, and utilization of mouse specific primers.
Mycoplasma contamination	No mycoplasma was detected.
Commonly misidentified lines<br>(See ICLAC register)	None.

Animals and other research organisms

Policy information about studies involving animals; ARRIVE guidelines recommended for reporting animal research, and Sex and Gender in Research

Laboratory animals	Tumors were generated from male or female Ptch1+/-;K14-creER;p53f/f (with or without a RFP reporter). These mice were injected with tamoxifen at 7 weeks of age followed by UV irradiation. Approximately 6-8 months later, tumors would form making the mice 8-10 months of age at tumor collection. 7 week old female C57BL/6J mice (Jackson Laboratory stock number 000664) and 7-8 week old female NOD SCID (Jackson Laboratory stock number 001303) were used. Mice were housed at an ambient temperature of 72 degrees F with humidity of 42%, with a 12-hour light/dark cycle.
Wild animals	None.
Reporting on sex	Primary tumors were collected from male and female mice without any form of discrimination. No specific consideration was given to use of one particular sex over another. In transplant experiments, female mice were used solely on the basis of mouse cage consolidation (female mice could be housed with one another).
Field-collected samples	None.
Ethics oversight	All mouse usage (husbandry, procedures, experiments, etc.) were approved by the Institutional Animal Care and Use Committee (IACUC) at Stanford University.

Note that full information on the approval of the study protocol must also be provided in the manuscript.

Flow Cytometry

Plots

Confirm that:

☒ The axis labels state the marker and fluorochrome used (e.g. CD4-FITC).

☒ The axis scales are clearly visible. Include numbers along axes only for bottom left plot of group (a 'group' is an analysis of identical markers).

☒ All plots are contour plots with outliers or pseudocolor plots.

☒ A numerical value for number of cells or percentage (with statistics) is provided.

Methodology

Sample preparation	As noted in methods section, mouse tumors were briefly rinsed with 1x PBS before being chopped and minced into pieces less than 1 mm in diamter. After mincing, tumor pieces are transferred to 50-mL conical tubes and 40 mL of 0.5% collagenase (Gibco; 17-100-017) solution in DKFSM media (Gibco; 10744-019). The minced tumors are then incubated within the solution at 37deg C with rotation for 1 hour. After 1 hour, 5 mL of 0.25% Trypsin (Gibco; 25200056) was then added to the 50-mL conical tube and further incubated for 15 minutes. 5 mL of FBS was then added to the cellular suspension. Cells were filtered with a 70-µm filter before being pelleted. Cells were either immediately used or frozen down in BAM Banker (FujiFilm; CS-02-001). Human tumors were processed in the same manner except that they required a 2 hour collagenase digestion as compared to mice, which only need 1 hour. For isolation of normal mouse epidermis, mice were shaved, back skin removed, and underlying fat was scrapped off. The skin was then minced into pieces that were smaller than 1 mm in diameter. Minced samples were placed in 15-mL conical tubes and digested with 10 mL of 0.25% collagenase solution in DKFSM. Samples were incubated at 37°C with rotation for 2 hours. 1 mL of FBS was then added to the cellular suspension. For all flow experiments, samples were re-suspended in a 2% FBS for staining.
Instrument	For flow analysis, we used the LSRII instrument and for sorting, we used the FACSAria II.
Software	BD FACSDiva software was used for data collection on the instrument. For downstream analysis, we used FloJo v10.6.
Cell population abundance	For live/dead sorting for different human tumors, live cell fraction made up approximately 40-70% of the cellular populations. For mouse Ly6d+ and Ly6d- sorting or analysis from primary tumors, the Ly6d+ population made up around 15-30% of the tumor epithelium, depending on the tumor. For mouse Ly6d+ and Ly6d- analysis from organoids, the Ly6d+ population varied depending on the drug intervention from close to 0%-30%.
Gating strategy	FSC-A/SSC-A gating was first used to identify cells and remove debris. Single cells were isolated by SSC-W/SSC-H and a subsequent FSC-W/FSC-H gating. Live cells were then isolated via negative staining for SytoxBlue. For mouse samples, gating on RFP was done to identifying tumor epithelial cells before Ly6d gating. The boundaries between positive and negative staining were drawn based on a combination of using unstained controls as well as single stain controls.

☒ Tick this box to confirm that a figure exemplifying the gating strategy is provided in the Supplementary Information.
